# “Eco-caring together” pro-ecological group-based community interventions and mental wellbeing: a systematic scoping review

**DOI:** 10.3389/fpsyg.2024.1288791

**Published:** 2024-04-24

**Authors:** Kane Baker, Bianca Chioran, Elizabeth Marks

**Affiliations:** Department of Psychology, University of Bath, Bath, United Kingdom

**Keywords:** nature-based interventions, pro-environmental behavior (PEB), conservation, social integration, mental health, pro-social behavior, climate anxiety

## Abstract

**Background:**

Poor mental wellbeing is a challenge for societies across the world, as is the increasing threat of climate change, and emerging evidence suggests these challenges are interrelated. Green and social prescribing of non-clinical interventions hold promise as a cost-effective and widely accessible way to improve wellbeing, and interest is growing in whether pro-ecological communal activities have mutual benefits for both people and the planet.

**Objectives:**

Communal pro-ecological activities are growing in popularity, and research is gathering pace into whether participation influences mental wellbeing. The present systematic review scopes the existing evidence base to explore what is being done, what is being found, and what additional research is required.

**Methods:**

Electronic databases (PsychNET, PubMed, Scopus, and Web of Science) were searched for studies that involved groups of people undertaking pro-ecological activities, where components of mental wellbeing were assessed. Eligibility criteria were purposely broad, including all study designs and participants across the lifespan.

**Results:**

Thirty-seven eligible studies were identified. Nearly half of the studies used mixed-method designs, and most studies used surveys or interviews to evaluate outcomes. Most pro-ecological activities involved planting vegetation, and habitat creation, maintenance, or restoration. Methodological quality varied considerably. Among the perceived therapeutic mechanisms reported, the social elements of the interventions were prominent.

**Discussion:**

Coherent synthesis of the current evidence base is challenging given the heterogeneous range of methods, samples, and interventions within the studies. However, the results here demonstrate promise that with future research and better methodological rigor, pro-ecological group-based interventions hold the potential to improve mental wellbeing and influence sustainable behavior.

**Systematic review registration:**

https://osf.io/vmpr6/.

## 1 Introduction

Poor mental health and the climate and ecological crises are two of the most significant issues facing humankind globally (Lawrance et al., 2021). The disabling consequences of mental distress impact around one billion people worldwide (Institute for Health Metrics and Evaluation, 2017), and in the U.K., one in four adults in any given year experiences at least one diagnosable mental health condition (NHS England, 2021). Alongside this, the insidious adverse consequences of the changing climate and related ecological degradation and biodiversity loss, are progressively disastrous for humans (e.g., Kjellstrom et al., 2018). We are seeing increasing numbers of extreme weather events, meaning growing numbers of people are being directly impacted by climate change in a way that causes distress (Intergovernmental Panel on Climate Change, 2021; Lawrance et al., 2021).

In addition, there is evidence that even the awareness of the climate and ecological crises causes mental distress for many people (Pihkala, 2020). Variously described eco-anxiety, ecological grief, and solastalgia, involving the experience of painful emotional and cognitive responses to the climate crisis including anxiety, sadness, and anger, are growing in recognition (Clayton and Karazsia, 2020; Stanley et al., 2021). Rates of such eco-distress are increasingly significant, particularly in younger populations (Hickman, 2020). Although not a mental illness (e.g., Lawton, 2019), distress related to the climate and ecological crises is a chronic and inescapable stressor that may increase vulnerability to other mental health problems (e.g., Zuckerman, 1999).

As such, there is growing evidence that the climate and ecological crises have a significant, complex, and evolving impact on human mental health and wellbeing (Lawrance et al., 2021). This relationship is complicated by findings that, in contrast, pro-environmental behavior and connection to nature are associated with subjective wellbeing (Chukwuorji et al., 2017). This indicates how such issues are intertwined, and with the deterioration of the natural world, the mental health difficulties of global populations will inevitably grow more problematic (Romeu, 2021). For example, a correlating factor is the rising rate of modern urbanized living, which can have negative impacts on mental health through the increased isolation, loneliness, and disconnection such lifestyles can create (Zijlema et al., 2015; Klussman et al., 2020). Alongside this, the loss of natural environments may limit how much access many people have to nature and its positive wellbeing impacts.

The gap that exists between mental distress and effective treatment (Wainberg et al., 2017) woefully resembles the gap seen between climate change and mitigative action (Intergovernmental Panel on Climate Change, 2021). In England and Wales, only one in eight people receive treatment for their mental health problem, most commonly, medication (McManus et al., 2009, 2016; Welsh Government, 2016). For children, where mental health difficulties can have dramatic long-term consequences, only a quarter with a diagnosed psychiatric disorder had contact with a mental health specialist in 2017, and over 20% of these children waited longer than 6 months (Sadler et al., 2018). It is thus essential to develop novel, scalable, efficient, and timely interventions, while improving social support networks to tackle this increasing mental health burden, particularly considering the context of limited funding for services.

Green-prescribing is an umbrella term for nature-based non-clinical interventions that are offered to people to alleviate distress and improve their mental wellbeing (Leavell et al., 2019). Several theories explain why nature contact is beneficial. The Biophilia Hypothesis (Wilson, 1984) suggests that the human brain evolved in a biocentric world, attuning it to extract, process, and evaluate information in the natural environment. As such, humans have a genetically predisposed attraction to nature, for which we seek connection with (Wilson, 1993). Moreover, Habitat Selection Theory (Orians and Heerwagen, 1992) poses those natural surroundings aided survival through evolution, and as such, attraction to them is cross-cultural and universal. Such theories, however, may oversimplify the complexity of nature-wellbeing links in a rapidly modernizing and changing world (e.g., Joye and De Block, 2011). Nonetheless, simply spending time in nature can be beneficial for mental wellbeing, and it has been suggested that this is through cognitive restoration and reducing stress (Bragg and Atkins, 2016; Richardson et al., 2021). Indeed, Attention Restoration Theory (Kaplan and Kaplan, 1989) suggests that exposure to nature can replenish concentration and reduce mental fatigue, and Stress Reduction Theory (Ulrich et al., 1991) poses that looking at natural scenery can improve emotional and physiological states, aiding emotional regulation through an involuntary reduction of arousal.

Social prescribing is another umbrella term for non-clinical interventions that promote group community-based activities, which are also considered beneficial for mental health (e.g., Esmene et al., 2020). The Main Effect Model (Rook, 1990) suggests that social integration provides regular positive and rewarding experiences that bolster feelings of security, purpose, and belonging, whereas the Stress-Buffering Model (Cohen and Wills, 1985) suggests that social relations can promote our perceived ability to cope with imposed life stresses. Though such theories can overgeneralise the complexity of social interaction and relationships across individuals and cultures (e.g., Landerman et al., 1989), studies from the realm of community psychology generally show that a sense of responsibility, belonging, and cohesion within a neighborhood is linked to mental wellbeing (Sarason, 1974; Elliott et al., 2014). Given that social disconnection and loneliness predict mental distress (Klussman et al., 2020), social prescribing makes sense, and networking among people with shared experiences, concerns, or disorders can be therapeutic (e.g., Isaksson et al., 2021).

A shared intent, or perhaps a by-product of green and social prescribing, is encouraging physical activity to promote mental wellbeing (Chatterjee et al., 2018). Various biological and psychological theories describe this link. For instance, the Endorphin Hypothesis (Hoffman, 1997) states that exercise increases the release of β-endorphins that stimulates positive mood, whereas the Self-Efficacy Hypothesis (Craft, 2005) suggests that exercise provides a meaningful mastery experience that can boost self-belief and confidence.

With theory and evidence highlighting the health benefits of being in nature and joining community activities, there is a strong argument for combining green and social activity prescribing. Participating in nature-based group activities could offer particularly powerful benefits (Fixsen and Barrett, 2022), and evidence supports this claim. For instance, group nature walks are shown to have greater health benefits than group walks in urban areas or walking alone (e.g., Marselle et al., 2013; Hanson and Jones, 2015).

However, green social prescribing interventions have the potential to go even further; by moving from *“being in”* to *“doing with”* nature. Pro-ecological group-based activities such as communal tree planting have great potential for positive mental health impacts as they incorporate multiple elements, each offering particular benefits. This includes exposure to nature, social connection, and exercise as discussed, but also implicates pro-social and pro-environmental behavior (e.g., O’Brien et al., 2010; Taufik et al., 2015; Martin et al., 2020). Such behaviors, involving actions intended to benefit others or the environment (e.g., Penner et al., 2005; Steg and Vlek, 2009), are beneficial for mental wellbeing cross-culturally (Hui et al., 2020; Capstick et al., 2022), and a new reciprocal model proposes that a positive feedback loop exists between prosocial behavior and mental health (Hui, 2022). Response Shift Theory (Schwartz and Sendor, 1999) poses that pro-social behavior can shift internal values to help people realize the meaning and value of life and distract them from their own worries and stress. Similarly, the Negative-state Relief Model (Cialdini and Kenrick, 1976) suggests that pro-social action helps to reduce negative mood, whereas the Warm-Glow Theory focuses on the experienced joy and satisfaction people gain from doing good for others or the environment (Andreoni, 1989; Hartmann et al., 2017).

As people become increasingly concerned and interested in current threats to the natural world, pro-environmental behavior may be of particular importance. Empowerment Theory (Perkins and Zimmerman, 1995) is thus relevant here, suggesting that people can gain self-esteem, self-efficacy, and internalized locus of control through collective action and group participation involving mutual respect, caring, and reflection to achieve goals. Evidence already indicates that pro-ecological collective action can promote wellbeing components, such as active hope, in those experiencing eco-related distress (e.g., Nairn, 2019; Stanley et al., 2021).

With multiple hypothesized processes at play, it is unlikely that any single theory can capture what might be happening in these activities. Nonetheless, *“eco-caring together”* interventions are receiving greater attention as they offer benefits for human mental health and planetary health (Robinson and Breed, 2019; Breed et al., 2020). Given the influences of collective issues such as loneliness and eco-distress on mental suffering, focusing treatments and responses upon the individual could be seen as contradictory, while more community-focused remedies seem increasingly appropriate. Despite such promise, the shape and extent of the literature investigating pro-ecological group-based interventions are unclear, possibly due to the heterogeneity of such interventions and assessment methods. This systematic review aims to scope the state of the literature on studies that have explored pro-ecological, group-based community activities and their influence on mental wellbeing.

To our knowledge, the only review of environmental enhancement interventions and their links to human health conducted was by Husk et al. (2016). Their searches in 2012 found 19 eligible studies, which were widely heterogeneous in terms of samples, designs, and evaluation methods. There was a strong risk of bias as the majority of studies were program evaluations funded by intervention providers. The authors concluded that little quantitative evidence exists showing the benefits, but qualitative data showed some perceived benefits. The review was very broad, attempting to synthesize evidence from both group and *n* = 1 studies for both mental and physical health. Additionally, some activities included elements that were arguably not *pro*-environmental, but rather, aesthetic work in nature (e.g., pathway creation), and the review excluded activities such as communal gardening. Nonetheless, a main conclusion was the inherent difficulty with generating robust evidence for such interventions, and recommendations for linked reviews to refine the understanding of environmental interventions were made.

This review comes a decade on from Husk et al. (2016) searches, at a time when understanding and concern around environmental issues are much greater, among both the public and researchers. The current review employed a refined scope, searching for literature relating to *group*-*based* interventions, with *pro-*ecological elements, that focussed on *mental* wellbeing. Six research questions guided this review to explore what the evidence base now looks like, shedding light on whether it is time to conduct a more definitive systemic review or meta-analysis:

1. What is the current state of the evidence base for pro-ecological, group-based activities to promote mental wellbeing? *(Primary Question)*
2. What study designs are used to evaluate such interventions?
3. How are such interventions evaluated, and what key outcome measures are utilized?
4. Are there indications of the perceived therapeutic mechanisms of such interventions?
5. Are there indications of the acceptability and challenges of such interventions?
6. Are there any indications about how such approaches are experienced by people reporting eco-related distress?

## 2 Materials and methods

### 2.1 Protocol and registration

The Preferred Reporting Items for Systematic Reviews and Meta-analyses (PRISMA) guidance 2020 (Page et al., 2021) and extension for scoping reviews (PRISMA-ScR) (Tricco et al., 2018) were followed. The protocol was registered on the Open Science Framework^1^ before searching began.

### 2.2 Eligibility criteria

The criteria were set broadly without date restrictions with an aim to capture the anticipated heterogeneity of relevant literature. Studies were included if they reported adult or child participants (with or without reported mental distress) involved in a group activity or intervention that contained pro-ecological elements, and where outcomes included at least one measure or report of mood, affect, life satisfaction, mental health, or wellbeing. Studies were excluded if they focussed solely on physical health, or if pro-ecological experiences were via paid employment. All study designs were included. Articles that were not in or translatable to English were excluded.

### 2.3 Search strategy and information sources

The search strategy was built to find studies that met four broad search targets: (1) pro-ecological activities/natural environments, (2) involving group/community participation, (3) reporting interventions/volunteering trials, which (4) included wellbeing/mood measurement (see Supplementary Appendix A). Search terms were generated using relevant literature to explore existing descriptors of pro-ecological actions and settings. In this review, “pro-ecological” refers to interventions that actively involve elements of green, sustainable, or eco-friendly behavior that have protective or enriching actions toward biodiversity or the environment. Synonyms for “group” and “intervention” were applied, and descriptors relating broadly to mental wellbeing were sourced through MeSH terms.

A systematic search of PsychNET, PubMed, Scopus and Web of Science databases was conducted on 26th January 2022. The search terms were applied to the title, abstract and keywords (for all database-specific search terms and limit filters, see Supplementary Appendix A). Reference lists from eligible studies were skimmed to identify further eligible studies. Generic online searches were completed on popular search engines to scope relevant gray literature and third-party organization reports. Finally, the WHO’s International Clinical Trials Registry Platform (ICTRP; trialsearch.who.int) and ClinicalTrials.gov (clinicaltrials.gov) were consulted for completed unpublished trials, using the search term “nature-based” OR “pro-ecological” AND “wellbeing” to maximize sensitivity (Hunter et al., 2022).

### 2.4 Source selection and management

Following database searches, records were managed using Covidence (2022) software. Duplicate records were immediately removed. At screening stage one, the primary reviewer (KB) independently screened all titles and abstracts for further full-text review eligibility. At stage two, full-text records were independently double-screened by the primary and secondary reviewers (KB, BC) for inclusion. Discrepancies were resolved by discussion between the two reviewers, and the senior author (EM) was consulted where consensus was not reached.

### 2.5 Data items and charting

The data-charting form developed by the lead author (KB) was trialed by both reviewers (KB, BC) and refined. Data of interest were guided by the research questions; primarily, intervention types, summary findings, study designs, evaluation methods, acceptability findings, and reported therapeutic mechanisms. Additional data extracted included: sample recruitment, size, and characteristics, publication type, and country of origin. Data from the included studies was charted by either reviewer (KB, BC) using Covidence (2022) software and then cross-checked by the other to ensure consistency and accuracy.

### 2.6 Critical appraisal

The Mixed Methods Assessment Tool (MMAT) (Pluye et al., 2009; see Supplementary Appendix C) was used to evaluate the quality of included studies, enabling assessment of both the state and strength of the evidence base. The MMAT is a single integrated tool that allows the assessment of quantitative, qualitative, and mixed-methods studies. Two screening questions were initially applied to each study; (i) is there a clear research question? (ii) does the data collected address this question? Studies failing these questions, or not reporting any outcomes, were not suitable for the MMAT. Studies meeting criteria were further assessed using five (“yes”/”no”) criteria relevant to the methodology used. The number of criteria met (scored “yes”) was calculated and reported as a quality score percentage (0, 20, 40, 60, 80, 100%), allowing comparison across methodologies.

### 2.7 Synthesis

Extracted data was exported to Microsoft Excel for analysis. An overarching narrative synthesis was most appropriate given the heterogeneity of included studies. The synthesis and reporting were structurally guided by the research questions. Where appropriate, descriptive quantitative analysis was applied to charted data, giving frequency and percentage results.

## 3 Results

### 3.1 Sources of evidence

**Figure 1:**
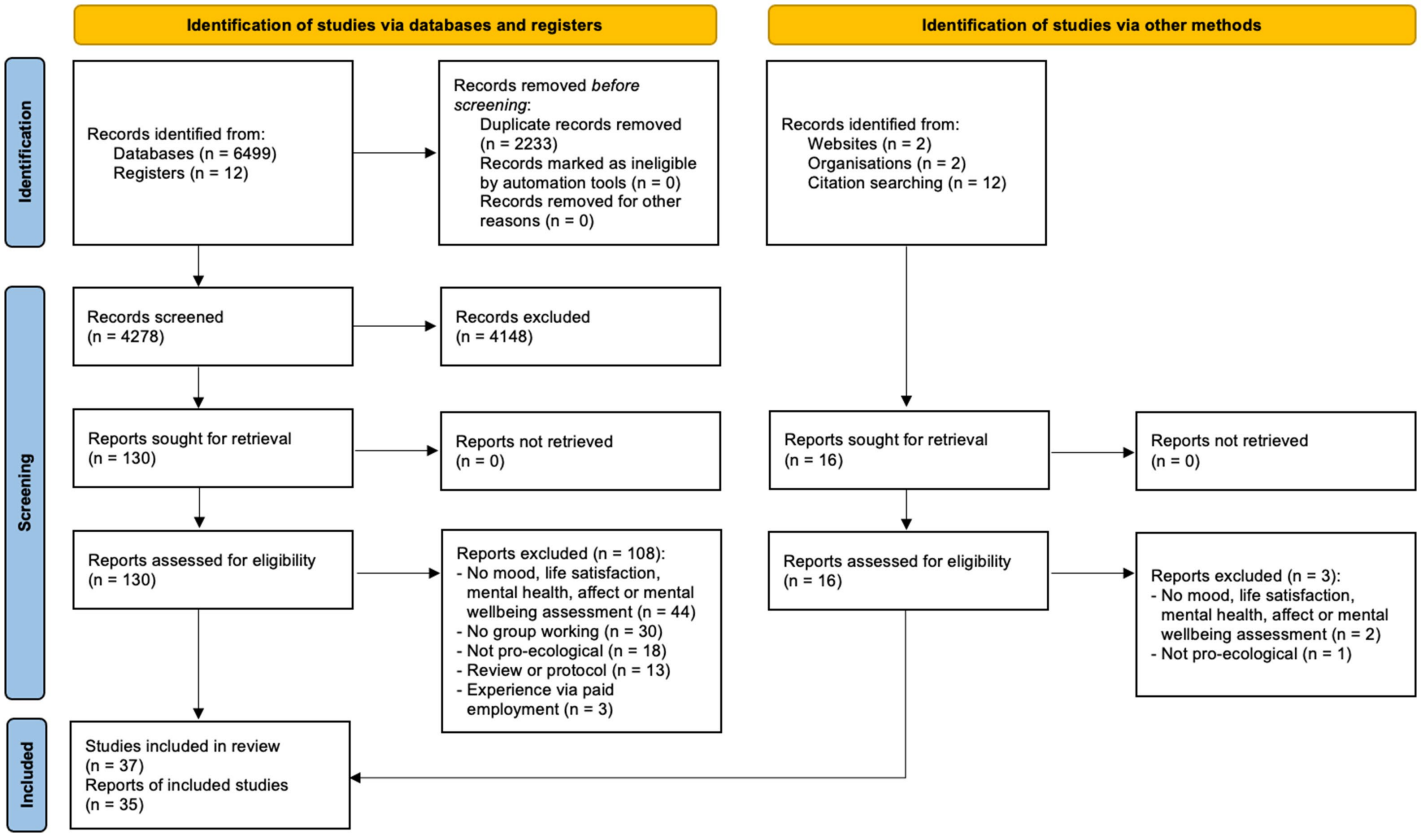
PRISMA flow diagram (Page et al., 2021).

The process of study selection is shown in Figure 1. The search identified 6,499 records from electronic databases, 12 records from registers, and a further 16 papers and reports were identified from other sources. After duplication removal, 4,278 papers were title and abstract screened, and 146 papers were included in the full-text screen (see Supplementary Appendix B for excluded papers and reasons). A total of 37 studies from 35 papers were eligible for inclusion (Townsend, 2006; contained three distinct eligible studies).

### 3.2 Review question 1: Current state of evidence base (including characteristics of sources)

Data from 37 studies were charted from 35 individual papers, and key characteristics are shown in Table 1. As anticipated, the range of studies was varied and diverse, reflecting the broad scope and inclusion criteria of this review. The earliest study found was from 1998, with gradually growing interest in pro-ecological activities and mental wellbeing published since.

**Table 1:** Characteristics of sources.

Study (author, date; country)	Design (method; instruments)	Sample (n; characteristics; recruitment)	Pro-ecological intervention (program; activities [joint therapy]; duration)	Evaluation (time-points; measures)	Perceived therapeutic mechanisms	Key findings
Asah and Blahna (2013); USA	Mixed methods; interviews and online surveys	242; conservation group volunteers, aged 18–81, 80% white, 66% female; opportunity sampling	Volunteer-dependent urban conservation events; specific activities and duration not reported	Post-; online surveys developed from initial interviews	Making a difference; enjoyment; connecting with community; sense of belonging; social interaction	Motivations to volunteer include more satisfaction and sense of social belonging by allowing individuals to give back to the community and connect with other like-minded people
Avon Wildlife Trust (2021); UK	Mixed methods; interviews and psychometric tools	74 (*n =* 22 follow-up); conservation group volunteers, adults; purposive sampling	Green care; food growing, seed sowing, pond dipping, and creation of new meadow, insect hotel, pollinator beds, bird feeders; up to 18 weekly sessions; 4.5 h each	Pre-, Post-, 2y Follow-up; interviews, Holistic Health and Nature Scale, Pro-Nature Conservation Behavior Scale	Being outside in nature; social interaction; learning about using nature to alleviate stress	89% improved wellbeing (e.g., confidence, motivation, mood); 78% increased community connection; Self-reported decrease in stress, anxiety, and panic, calmer and more present, can better cope with problems; maintained wellbeing at follow-up
Bellotti et al. (2011); USA	Quantitative, pilot cohort study; psychometric tools	17; unemployed veterans, aged 24–57, 15 male; purposive sampling	Green jobs training program; wastewater management, recreation enhancement, habitat restoration; 2 days a week, 10 months	Pre-, Mid-, Post-; Beck Depression Inventory, Beck Anxiety Inventory, PTSD Checklist, Short Form Health Survey, Quality of Life Inventory	Peer support; spending time outdoors	Depression, anxiety, and PTSD reduced but not significantly. Significant increases in physical and social functioning.
Birch (2005); UK	Qualitative; observations, interviews, and notebook analysis	3; conservation group volunteers, aged 39–62, 2 female; opportunity sampling	Green gym; clearing brambles, preparing soil for nature garden, creating vegetable plots, planting trees, woodland rubbish clearance; 3 h weekly 10–14 weeks	Mid-; participant observations, semi-structured interviews, photographs taken by participants	Participating in achievable, tangible, and socially valued work; enjoyment; stimulation, sense of achievement; social teamwork	Positive effects on mental wellbeing, improved mental state, reduced stress, increased sense of achievement
Bond et al. (2019); AUS	Mixed methods, cross-sectional study; online surveys	2,453; Trees for Life non-profit organization members; purposive sampling	The tree scheme; volunteers grow seedlings for revegetation on rural land; duration not reported	Post-; online surveys to measure perceived benefits of the scheme	Teamwork; engaging with others; satisfying work; helping others, enjoyment, urban–rural connection	78% of volunteer growers reported participation as personally satisfying (including receipt of social benefits from participation, helping landholders, and working with family or others in a team) and enjoy the process of growing seedlings and developing their own capacity (knowledge and skills) through the activity
Cardskadden and Lober (1998); USA	Mixed methods, case report; mail surveys	85; corporate employee volunteers; purposive sampling	Corporate wildlife habitat enhancement program; tree planting, creating nest boxes and wildflower meadows, wetland, and upland restoration; 13 h	Post-; mail surveys to measure perceived benefits of the program	Enhanced community contact	Program improved employee morale, pride, promoted self-motivation, and helped build stronger social (e.g., with community) relationships
Coventry et al. (2019); UK	Mixed methods, pilot study; interviews, surveys, and psychometric tools	45; conservation group volunteers, mean age 43.8, 59% male; opportunity sampling	Conservation volunteering; flood mitigation, scything, pruning, and creating wildlife habitats; 20–30 min	Pre, Post-; interviews, Short Warwick-Edinburgh Mental-Wellbeing Scale, Acute subjective Mood Adjective Checklist	Sense of belonging; social interaction; sharing aspirations; purposeful and meaningful work	Conservation work led to improvements in subjective wellbeing, mood, and stress. Work was perceived as purposeful and meaningful. Significant reduction in stress and increase in hedonic tone.
Finnegan (2016); UK	Qualitative; interviews	14; veterans with mixed physical disabilities, aged 23–62; 86% male; purposive sampling	Defense archeology group; surveying; geophysics and ordnance recovery; 10–14 days	Post-; interviews to measure perceived benefits and detrimental effects of the scheme	Sense of achievement; socialization opportunities; sense of appreciation	Veterans voiced improved self-esteem, self-worth, mood, and feelings of achievement
Fraser et al. (2009); USA	Qualitative; surveys and interviews	51; retired zoo volunteers, aged 55–76, most Caucasian; purposive sampling	Zoo volunteering; promoting conservation to visitors, conservation activities; duration not reported	Mid-; surveys and interview to measure motivations, experiences, and satisfaction with volunteering	Connection with nature; sense of self-purpose; value-expression	Participants reported improved self-esteem, gaining a sense of meaning and purpose, extending their social identity, and personal affirmation
Gagliardi et al. (2020); Italy	Mixed methods; focus groups and psychometric tools	19; mean age 75.7, 11 male, 68.4% lower education level; voluntary sampling	Civic environmental volunteering program; cleaning up areas, removing twigs along the trails, reporting hazards, maintenance and repair work in damaged areas or equipment; twice weekly session for one year	Pre-, Post-; focus groups, Positive and Negative Affect Schedule, Life Satisfaction Measure, Lubben Social Network Scale	Social factors; social bond formation; interacting with others; sense of belonging; making friends; being part of a group; relaxation	Positive variations were found in life satisfaction, affect and feelings of social support. Feeling of distress significantly decreased, alongside an increase in positive feelings. Participants reported making new friends, being a part of a group, getting one’s mind off things, relaxing, having a sense of purpose, positivity, calmness, improved self-esteem. Reports of wider family noticing mood improvements.
Gooch (2005); AUS	Qualitative; interviews	85; catchment group volunteers; purposive sampling	Catchment volunteering; natural resource management along the coast to combat land degradation; duration not reported	Mid-; interviews to measure experiences of the volunteering	Personal attachment; enjoyment; sense of belonging; sense of purpose; working toward a common goal	Volunteers felt empowered, balanced, and satisfaction. They expressed deep levels of satisfaction and felt valued by others in the group.
Hoffman (2020); USA	Mixed methods; surveys	17; 10 inmates, 7 community members; voluntary sampling	Community reintegration; orchard creation, tree planting; 5 h	Post-; survey to explore experiences	Social connectedness; feelings of belongingness; being outdoors; physical activity; enjoyment; building something positive for the community	Helped inmates with their spiritual and mental ability. Some reported it to be therapeutic. Perceptions of belongingness and connectedness to community also increased. Community members gained a better understanding of the inmates.
Hsiao et al. (2020); Taiwan	Quantitative, quasi-experimental longitudinal study; psychometric tools and medical records	72 (*n =* 36 recycling volunteers, *n =* 36 controls); mean age 71.5, 75% female; purposive and voluntary sampling	Tzu Chi recycling program; voluntary collecting, sorting, reclaiming reusable resources at recycling stations, making eco-friendly blankets for disaster survivors; at least 1 day per week	Pre-, Post-; Self-Compassion Scale, Compassion Scale, Geriatric Depression Scale, Chinese Hostility Inventory, Chinese Happiness Inventory, medical records	Working together to achieve a goal; sense of belonging; social inclusion; shared humanity; sense of achievement; expanding social circle; outdoor space and connection	Intervention had significant long-term effects (1 year period) in improving self-compassion and compassion for others, and happiness levels. Significant reductions in negative outcomes such as depression, hostility affect, and hostility suppression. Intervention helped build resilience for coping with life adversities. Control group reported decreased levels of self-compassion and happiness and maintained levels of depression.
Kogstad et al. (2014); Norway	Qualitative; interviews	9; non-school or work attending, aged 17–27; purposive sampling	Green care; weeding vegetable gardens, attending to and feeding animals; 2 months to 2 years	Post-; interviews to measure elements and achievements of the volunteering	Group atmosphere (safe, kind and honest); silence and acceptance in natural environments; not being judged	Reports of improved self-esteem, confidence and intervention helping with mental health problems.
Koss (2010); AUS	Mixed methods; surveys	271; sea search volunteers, aged 18–71; mostly male; purposive sampling	Sea search volunteer program; teaches volunteers how to collect marine biota data using scientific methodologies, collected data contributes to coastline protection; duration not reported	Mid-; surveys to measure experiences, enjoyment, and wellbeing	Practical activity; outdoor activity; meaningful work; enjoyment; opportunity to socialize; belonging to a group; connecting with other like-minded people; sense of achievement	Volunteers gained a significant sense of enjoyment and achievement from participating and all strongly agreed that the activities generated personal satisfaction. Volunteers agreed that they felt good emotionally and mentally from carrying out marine activities.
Molsher and Townsend (2016); AUS	Mixed methods; interviews and psychometric tools	32; aged 14–72, 16 female, 10 unemployed, 5 with MH difficulties; purposive sampling	Get dirty feel good program; cockatoo recovery, koala management, seagrass monitoring, dolphin surveys, sea lion conservation, marine debris collection and weed management; 9–10 weeks, 5 h per week	Pre-, Post-, 3 m Follow-up; interviews explored experiences, General Wellbeing Scale, Emotional State Scale	Group dynamic; learning skills; social connectedness; work to help	Participants experienced positive emotional shifts during activities (>60% of mood parameters improved). General wellbeing significantly improved and tended to remain high at follow-up.
Moore et al. (2006); AUS	Mixed methods; interviews, surveys, and psychometric tools	102 (*n =* 51 community land management volunteers, *n =* 51 controls); most aged 45–64, 61 males; purposive and voluntary sampling	Community land management; protecting biodiversity, preserving native flora and fauna; average membership 7 years	Mid-; interviews to assess wellbeing, surveys to assess anxiety and depression, Community Cohesion Scale	Increased social networks; feelings of belonging; enjoyment; developing skills	Land management volunteers reported experiencing higher levels of mental wellbeing than controls. Many indirect benefits reported by volunteers, such as pleasure, enjoyment, and a sense of belonging to one’s community.
O’Brien et al. (2010); UK	Mixed methods; interviews, surveys, and psychometric tools	88; aged 16–76, 91% white British; purposive sampling	Environmental volunteering program; tree planting, vegetation clearance, removal of invasive species, sapling removal, tree thinning; 3 weeks	Pre-, Post-; interviews to measure experiences of volunteering, survey to assess wellbeing, Emotional State Scale, Personal Wellbeing Index	Contributing meaningfully to local community; building social networks; physical activity; being outdoors; sense of achievement; mental stimulation	Volunteers reported reductions in stress and mental fatigue and experienced a statistically significant positive emotional shift. Gained satisfaction with making a meaningful contribution to society and local communities. Found volunteering activities as therapeutic, mentally stimulating, and provided a sense of calmness and achievement. 21% experienced a negative change in general emotional states such as dissatisfaction and boredom.
O’Brien et al. (2011); UK	Qualitative; interviews	88; aged 16–76, 72% male, all white, mixed socioeconomic backgrounds, 25% marginalized; purposive sampling	Environmental volunteering program; general conservation activities; 10 h or less per month	Mid-; interviews to measure experiences of volunteering	Engaging in meaningful work; conserving nature; adding meaning to life; being outdoors in fresh air; enjoyment; learning; discovering new places	Volunteers reported improved wellbeing, improved relationships with others and family, better integration with community, and growing independence.
Pálsdóttir et al. (2014); Sweden	Mixed methods; interviews and psychometric tools	21; professionals with work stress; aged 29–68, 19 female, high education, homeowners; cluster sampling	Nature-based vocational rehabilitation; horticulture activities [alongside relaxing exercise, psychiatrist meetings]; 12 weeks, 3.5 h, 4 days per week	Pre-, Post-, 1y Follow-up; Stress and Crisis Inventory, Sense of Coherence Scale	Contact with nature; learning; creativity	Following intervention, participants reported significantly decreased stress. Contact with nature was reported as restorative and eased their minds.
Pillemer et al. (2010); USA	Quantitative, longitudinal cohort comparison study (1974–1994); surveys and psychometric tools	2,630 (*n =* 155 environmental volunteers); cohort mean age 44.7, 56.9% female; purposive sampling	Environmental volunteering; watershed monitoring, ecological restoration, environmental stewardship; 20 years	Mid-, 20y Follow-up; general wellbeing Likert scales and questions about environmental volunteering, Perceived Health Scale, Depression Scale	Not reported	Engagement as an environmental volunteer at baseline was significantly associated with reduced odds of perceiving oneself in fair or poor health, and reduced odds of experiencing depressive symptoms. Longitudinal analyses demonstrated a positive effect of both environmental volunteering.
Power and Smyth (2016); UK	Qualitative; focus groups and interviews	18; heritage conservation volunteers, aged 30s–70s, 14 females; purposive sampling	Preserving place; community heritage conservation, preserving local and historical assets from harm; duration not reported	Post-; interviews and focus groups exploring motivations, project origins, social impacts, and everyday experiences	Building new connections and friendships; socializing; learning	Intervention greatly benefited volunteers’ social wellbeing by helping them to build a wider social network and feelings of togetherness
Puhakka et al. (2019); Finland	Qualitative; surveys and interviews	74 (*n =* 49 parents, *n =* 25 day-care personnel) reporting wellbeing and play of children aged 3–5; purposive sampling	Green yards; children engaged in nature-based activities, looked after plants and vegetation, planted vegetables and flowers, watered forest floor mat and sod; 1 month	Post-; surveys and interviews exploring children’s activity, excitement, and wellbeing	Involvement in care; nature exploration; pleasant activities; time outdoors; physical activity; multi-sensory experiences; learning skills	The green yard activities had positive impacts on children’s and adults’ moods, wellbeing, energy, and motivation. Increased sense of community was reported by staff and children were enthusiastic to take care of plants.
Reynolds (2000); UK	Mixed methods; interviews and psychometric tools	15; aged 40–73, 8 male, 82% retired; voluntary sampling	Green gym; clearing overgrown vegetation, making room for rare species, building stiles, coppicing, planting trees, hedge laying; 2 months, twice a week	Pre-, Post-; interview about volunteering experiences, Medical Outcomes Trust Short Form	Meaningful work; being outdoors in the countryside; meeting other people	Volunteers reported improved quality of life, improved psychological wellbeing, and increased pleasure and satisfaction from doing something meaningful.
Richardson et al. (2016); UK	Quantitative; online surveys	126; aged 22–71, 15 males; voluntary sampling	30 days wild 2015 campaign; avoiding use of pesticides, leaving a patch of grass to grow long, alerting representatives to wildlife issues; 30 days	Pre-, Post-, 2 m Follow-up; survey exploring connection to nature, pro-nature behaviors, health, and wellbeing	Connection to nature; facilitating exercise; social contact; sense of purpose	Campaign found increases in participants connection to nature, and improved health, happiness, and wellbeing. Improvements sustained at follow-up.
Richardson et al. (2018); USA	Quantitative; online surveys and psychometric tools	380; mean age 49.5, 48 males, 93% white; voluntary sampling	30 days wild 2017 campaign; avoiding use of pesticides, leaving a patch of grass to grow long, alerting representatives to wildlife issues; 30 days	Pre-, Post-, 2 m Follow-up; survey exploring connection to nature, pro-nature behaviors, health, and wellbeing, Inclusion of Nature in Self Scale, Engagement with Beauty Scale, Difficulties in Emotion Regulation Scale	Connection to nature; noting natures beauty	Significant increases in wellbeing, in terms of happiness, were found from pre- to post-participation. Improvements in wellbeing were sustained at follow-up
Sobko et al. (2020); Hong Kong	RCT; surveys and psychometric tools	54 (*n =* 30 intervention, *n =* 24 controls); aged 2–5; voluntary sampling	Play&Grow program; children interacted with natural outside world, growing plants, caring for plants outside; 1 session per week, 10 weeks	Pre-, Post-; Children’s Stress Questionnaire, Connectedness to Nature	Connectedness to nature; interaction with nature	The intervention showed improved pro-social behavior and psychosocial wellbeing. Overall perceived stress significantly reduced, particularly anger frequency, among preschool children. Increased connectedness to nature following intervention.
Takase et al. (2019); Japan	Quantitative; online surveys	1,444; conservation volunteers, aged 20s–60s; 50% female; voluntary sampling	Conservation activities; duration not reported	Post-; survey exploring conservation experiences and motivations	Interaction with other people	Healing (improvement of mental wellbeing) rated as significant motivator (81% agreed). Social welfare rated as a key motivator (74% agreed). Improvement of mental wellbeing and wellbeing for local community, and interaction with other people also key motivators.
Tashiro (2022); Japan	Mixed methods; surveys and interviews	109; aged 18–80, 51% female, 50% unemployed; purposive sampling	Green management of rural region following post-disaster; planted camellia trees, picked up garbage, planted flowers along the road; duration not reported	Post-; survey and interview exploring perceived benefits, barriers, and green self-efficacy	Gifting future generations; social cohesion; helping local landscapes	Many agreed that participating in green activities promoted their self-worth. Green management induced hedonic experiences and promoted social cohesion.
Tharrey et al. (2020); France	Mixed methods; interviews, surveys, and psychometric tools	132 (*n =* 66 community gardeners, *n =* 66 matched controls), mean age 44, most female, educated; purposeful sampling	Community garden; caring for plants and plots; 8 months	Pre-, Post-; interviews exploring lifestyle changes, Warwick-Edinburgh Mental Wellbeing Scale, UCLA Loneliness Scale	Not reported	No significant statistical differences between participation and controls on mental wellbeing, social health, or connection to nature. Some participants reported strong or slight increases in life satisfaction and social relations.
Townsend (2006; Study 1); AUS	Qualitative; interviews	11; reserve management volunteers; purposive sampling	Friends of Damper Creek; management and maintenance of a small linear park; mostly weekends	Mid-; interviews exploring motivations and benefits of volunteering	Engaging in meaningful activities; building satisfactory relationships; nature contact; creative expression; positively contributing to society	Volunteers reported reduced stress, a greater sense of belonging and connectedness, and a widening of the social circle of children and families through membership. Mental and spiritual wellbeing from shared fun with others.
Townsend (2006; Study 2); AUS	Qualitative; interviews and surveys	18; residents volunteering to protect park from commercial sale; purposive sampling	Truganina explosives reserve preservation program; planning, developing and maintenance of nature reserve; mostly weekends	Mid-; interviews and surveys exploring involvement, health and wellbeing, and social capital and connectedness	Engaging in meaningful activities; building satisfactory relationships; nature contact; creative expression; positively contributing to society	Members perceived significant benefits relating to wellbeing: increased satisfaction stemming from doing something purposeful, mental relaxation, enjoyment, felt sense of accomplishments, and relief from outside pressures.
Townsend (2006; Study 3); AUS	Mixed methods; interviews, surveys, and psychometric tools	102 (*n =* 51 community conservation volunteers, *n =* 51 matched controls); purposive and voluntary sampling	Trust for nature; community-based conservation; protection of private land of high conservation value; mostly weekends	Mid-; interviews and surveys exploring, health, wellbeing, and social involvement, Neighborhood Cohesion Scale	Engaging in meaningful activities; building satisfactory relationships; nature contact; creative expression; positively contributing to society	Compared to controls, volunteers had perceived their health as better, and felt a greater sense of belonging in their communities.
Weston et al. (2015); Ghana	Mixed methods; focus groups and surveys	238 (*n =* 134 focus groups, *n =* 104 surveys with natural regeneration volunteers); purposive sampling	Farmer-managed natural regeneration; voluntary adding and managing trees and shrubs in farmlands or pastures; duration not reported	Post-; focus groups and surveys about the impact of the program on people’s lives	Optimism; community connection; community unity	Intervention boosted optimism of both participants and local community. It increased feelings of community unity. Improved psychosocial wellbeing (creation of aesthetically pleasing and comfortable environment), joy, and peace of mind stemming from intervention.
Wilkie and Michialino (2014); France	Quantitative; surveys and psychometric tools	73 (*n =* 43 community engaged residents, *n =* 30 non-engaged controls); mean age 47, 64% female; 58% unemployed; purposive sampling	Community engaged residents; pro-ecological activities, regeneration, or maintenance of public spaces; some regeneration projects done 10 years ago	Follow-up; surveys exploring life satisfaction, Personal Wellbeing Index, Social Cohesion Subscale	Social cohesion	Participative co-production had long-term benefits, including enhanced life satisfaction. Residents in areas with a strong participative co-production felt greater social cohesion.
Wilson et al. (2009); UK	Mixed methods; interviews, focus groups, and psychometric tools	77; aged 21–61, 20 females, all had history of MH problems; purposive sampling	Conservation program; removing unwanted tree seedlings, transplanting oak trees, construction using natural materials; 12 weeks	Pre-, Post-; interviews and focus groups explored experiences and attainments, Mental Component Summary Scale, SF-12 Health Scale, Warwick-Edinburgh Mental Wellbeing Scale	Engaging with nature; community engagement; social inclusion; meaningful work; sense of purpose; daily routine; leaning skills	Participants reported improvements to mental wellbeing through increasing confidence and self-esteem. No statistically significant difference between pre- and post-intervention on mental wellbeing scales.
Yerrell (2008); UK	Quantitative; surveys and psychometric tools	194; aged 25–64, 60% males, 71% unemployed; purposive sampling	Green gym; improving green spaces, gardening work, planting seeds, watering plants; minimum 3 months	Pre-, Post-; survey exploring motivations and benefits, Mental Component Summary Scale SF-12 Health Scale	Daily activity; exercise; being outdoors; improving the environment	Improvements in mental health following intervention. 99% either strongly agreed or agreed that improvements in mental wellbeing and confidence are key benefits gained from participating in the Green Gym. Personal achievement and positive self-worth reported.

**Figure 2:**
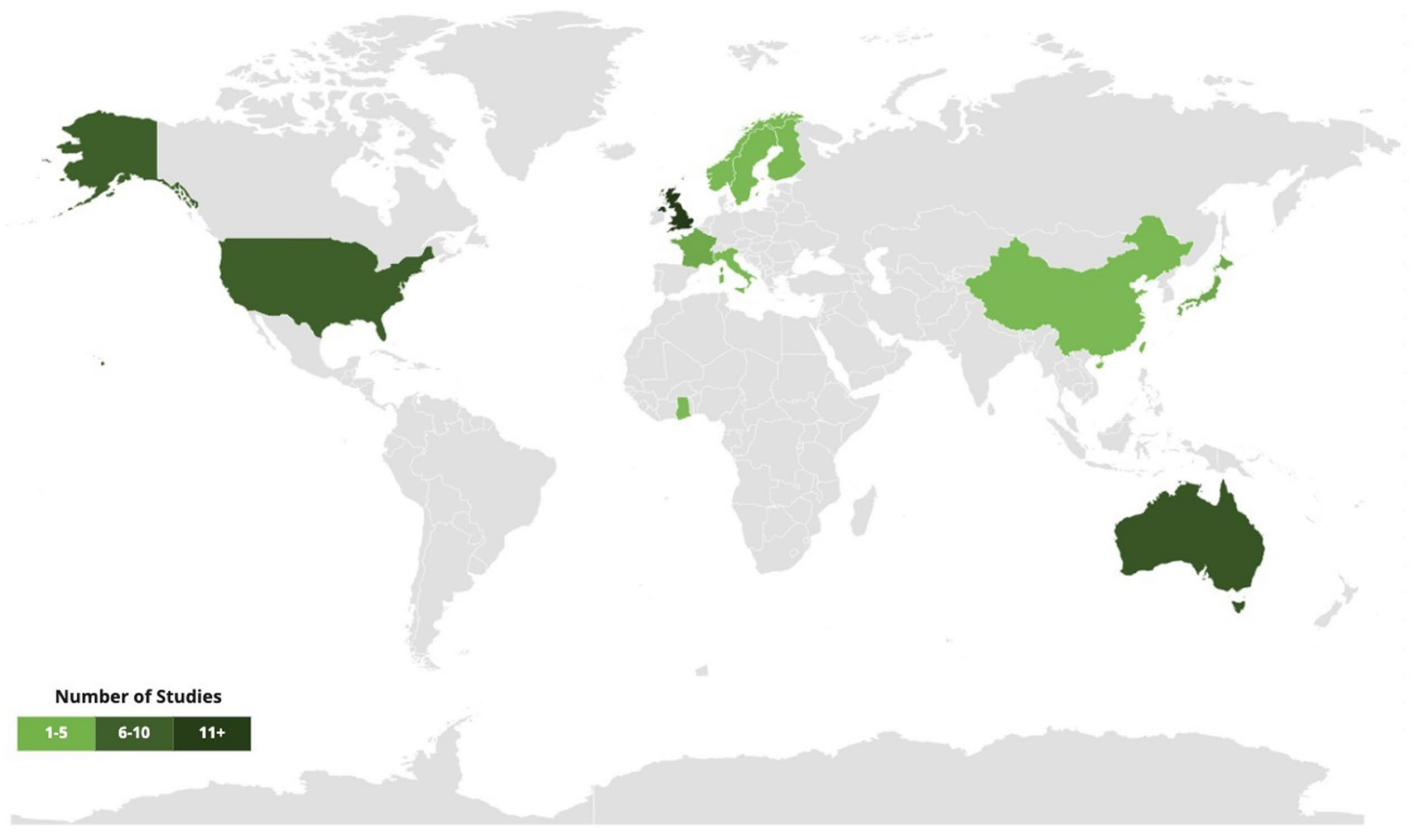
Study location.

In terms of location, studies were found across five continents, though most (46%) took place in Europe, many of which (30%) were in the UK (Figure 2). Very few studies reported participant ethnicity details.

**Figure 3:**
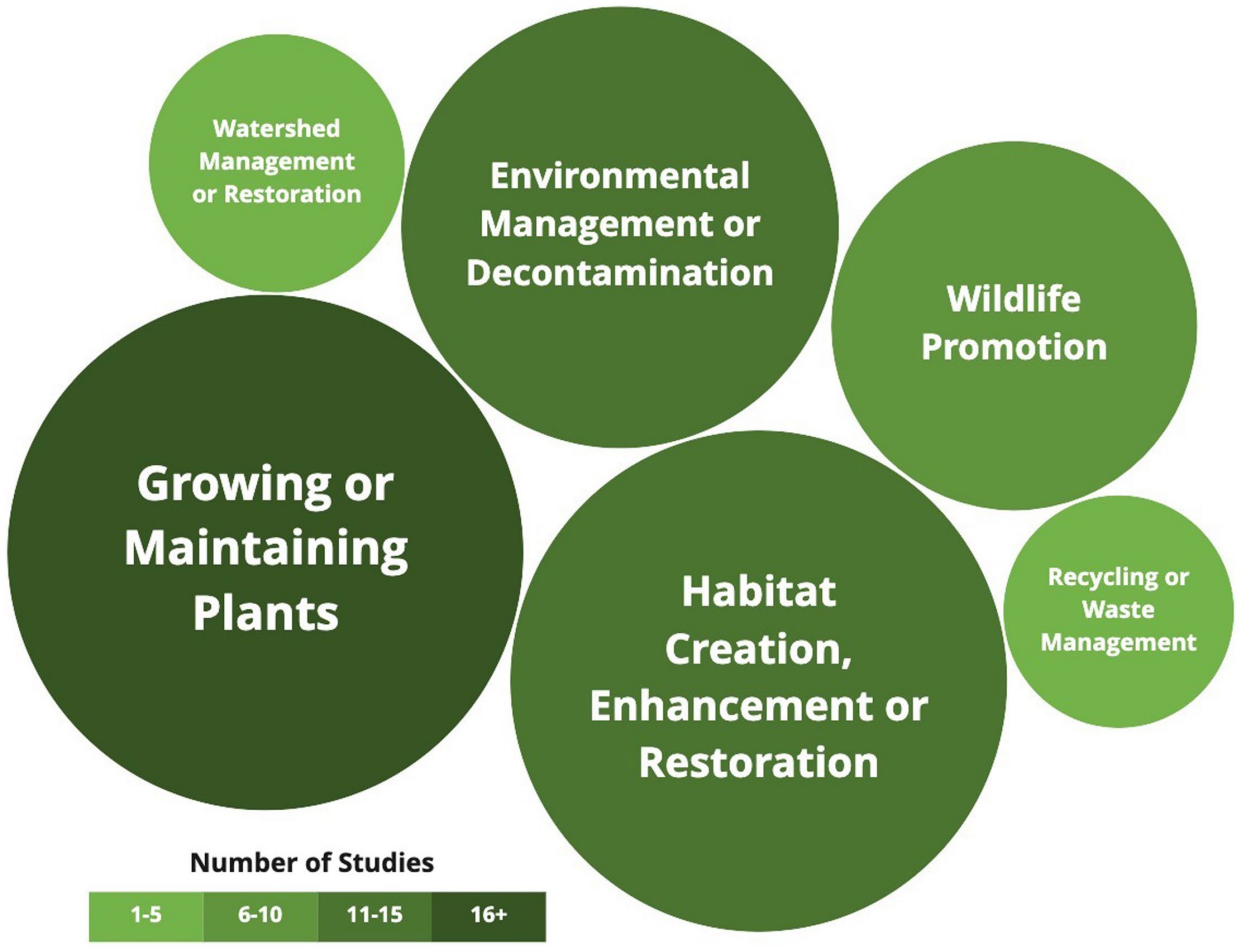
Pro-ecological activities.

As anticipated, the pro-ecological activities varied extensively across the studies. The range of different activities was broad, but all had pro-ecological behavioral elements. Common themes were found across the activities (see Figure 3), and some interventions included multiple activities that fell into several categories. The most common activities involved planting new trees or growing plants (43%), or habitat creation, enhancement, or restoration (41%). Environmental management or decontamination was reported in nearly a third of studies (32%), followed by wildlife promotion (24%). Watershed management or restoration (11%), and recycling or waste management (8%) activities were also reported.

Collectively, the studies sampled 9,483 participants, of which 2,733 were control or comparison participants. Sample sizes varied considerably, with some studies opting for in-depth evaluation of small groups (e.g., *n =* 3; Birch, 2005), to larger online evaluations of national campaigns (e.g., *n =* 2,453; Bond et al., 2019). Most studies (76%) reported the age range or average age of participants and included people across the lifespan, from as young as 2 years (e.g., Sobko et al., 2020) to 81 years old (e.g., Asah and Blahna, 2013). Most studies (68%) used purposeful sampling methods, with the rest using opportunity or voluntary recruitment methods.

Most studies (62%) assessed participants who were already undertaking the activities as part of a therapeutic or volunteering group program, a campaign, or as a hobby, with implications for sampling bias, although 38% engaged new participants in pro-ecological activities. Reporting of duration and timeframes spent on the activities varied widely (Table 1), with precise activity duration reported by better-controlled studies (e.g., “20–30 min”; Coventry et al., 2019), and other studies offering more vague estimates (e.g., “most weekends”; Townsend, 2006). For studies where participants were already undertaking the activities, few reported specific timeframes or duration of engagement (e.g., “an average of 7 years”; Moore et al., 2006).

### 3.3 Review question 2: Study designs (including critical appraisal of sources)

As shown in Table 2, study designs and methodologies were varied; mixed methods were most common (49%), followed by qualitative methods (27%), and then quantitative methods (24%). Most mixed methods studies (67%; *n* = 12) used surveys (four solely surveys, two with interviews, one with focus groups, and five with interviews and psychometric tools). The other six mixed-methods studies used interviews or focus groups alongside psychometric tools. All 10 qualitative studies used interviews, with three using qualitative surveys alongside, one using a focus group alongside, and one using observations alongside. Of the nine quantitative studies, six used descriptive methods, two used non-randomized group comparisons, and there was just one randomized controlled trial. Five quantitative studies used surveys plus psychometric tools, two used surveys only, and one used psychometric tools only.

**Table 2:** Study designs and quality assessments.

Study	Methods	Score	Criteria*				
Qualitative studies			1.1	1.2	1.3	1.4	1.5
Birch (2005)	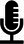 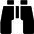	80%	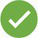	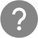	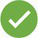	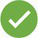	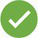
Finnegan (2016)	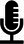	100%	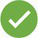	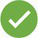	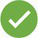	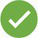	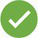
Fraser et al. (2009)	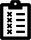 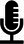	100%	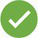	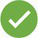	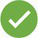	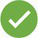	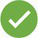
Gooch (2005)	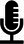	80%	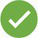	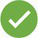	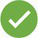	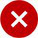	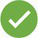
Kogstad et al. (2014)	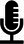	80%	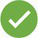	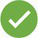	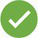	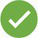	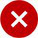
O’Brien et al. (2011)	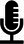	100%	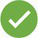	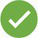	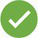	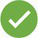	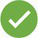
Power and Smyth (2016)	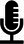 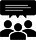	100%	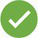	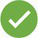	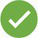	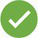	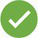
Puhakka et al. (2019)	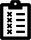 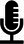	100%	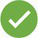	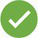	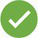	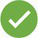	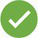
Townsend (2006; Study 1)	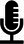	20%	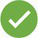	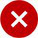	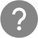	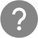	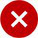
Townsend (2006; Study 2)	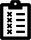 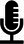	40%	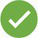	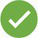	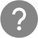	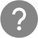	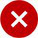
Quantitative randomized controlled trials			2.1	2.2	2.3	2.4	2.5
Sobko et al. (2020) 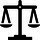	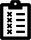 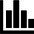	80%	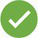	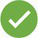	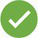	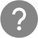	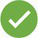
Quantitative non-randomized studies			3.1	3.2	3.3	3.4	3.5
Hsiao et al. (2020) 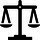	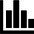	100%	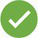	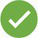	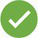	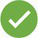	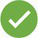
Pillemer et al. (2010) 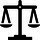	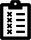 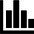	80%	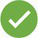	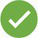	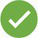	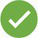	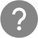
Quantitative descriptive studies			4.1	4.2	4.3	4.4	4.5
Bellotti et al. (2011)	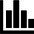	100%	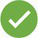	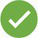	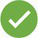	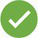	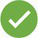
Richardson et al. (2016)	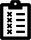	80%	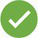	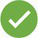	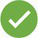	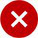	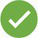
Richardson et al. (2018)	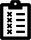 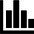	80%	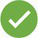	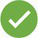	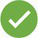	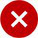	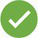
Takase et al. (2019)	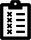	80%	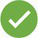	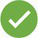	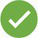	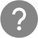	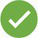
Wilkie and Michialino (2014) 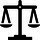	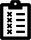 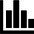	100%	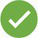	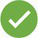	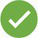	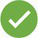	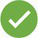
Yerrell (2008)	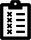 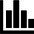	80%	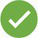	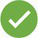	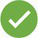	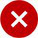	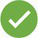
Mixed methods studies			5.1	5.2	5.3	5.4	5.5
Asah and Blahna (2013)	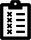 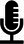	60%	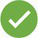	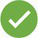	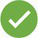	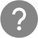	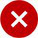
Avon Wildlife Trust (2021)	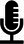 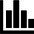	40%	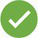	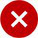	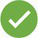	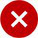	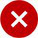
Bond et al. (2019)	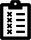	80%	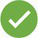	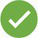	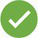	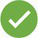	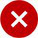
Cardskadden and Lober (1998)	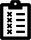	60%	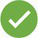	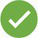	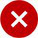	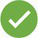	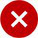
Coventry et al. (2019)	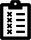 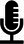 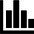	100%	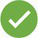	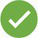	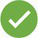	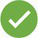	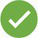
Gagliardi et al. (2020)	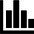 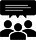	80%	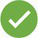	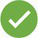	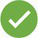	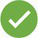	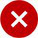
Hoffman (2020)	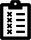	60%	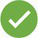	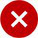	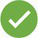	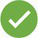	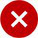
Koss (2010)	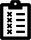	80%	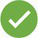	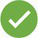	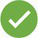	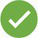	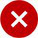
Molsher and Townsend (2016)	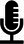 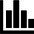	40%	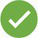	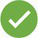	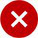	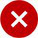	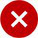
Moore et al. (2006) 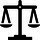	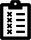 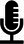 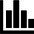	60%	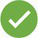	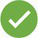	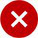	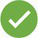	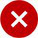
O’Brien et al. (2010)	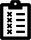 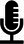 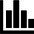	100%	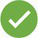	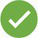	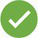	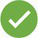	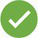
Pálsdóttir et al. (2014)	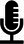 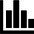	100%	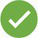	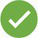	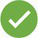	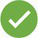	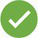
Reynolds (2000)	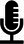 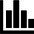	60%	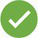	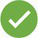	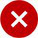	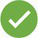	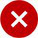
Tashiro (2022)	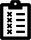 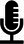	80%	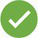	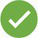	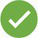	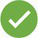	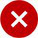
Tharrey et al. (2020) 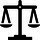	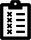 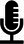 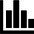	60%	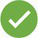	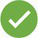	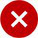	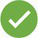	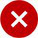
Townsend (2006; Study 3) 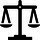	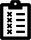 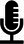 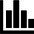	80%	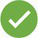	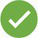	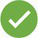	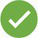	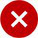
Weston et al. (2015)	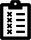 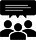	60%	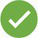	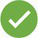	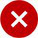	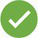	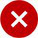
Wilson et al. (2009)	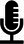 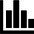 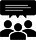	100%	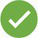	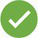	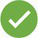	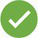	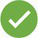

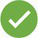
 met criteria;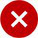 did not meet criteria;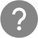 unclear if criteria met (*see Supplementary Appendix C for criteria);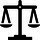 comparison study;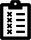 surveys;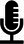 interviews;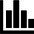 psychometric tools;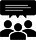 focus groups;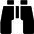 observations.

Only seven studies (19%) used control groups for between-group comparison, with two using matched controls. Only one purposely allocated matched participants to control or intervention conditions, where the intervention was not previously being undertaken by participants (Tharrey et al., 2020).

The MMAT quality assessment scores (Table 2) show higher scores for studies using quantitative (mean 87%) or qualitative (mean 80%) methods, compared to mixed methods (mean 72%). Overall, quality was highly variable with no clear association with the methods utilized.

### 3.4 Review question 3: Evaluation and outcome measures

**Figure 4:**
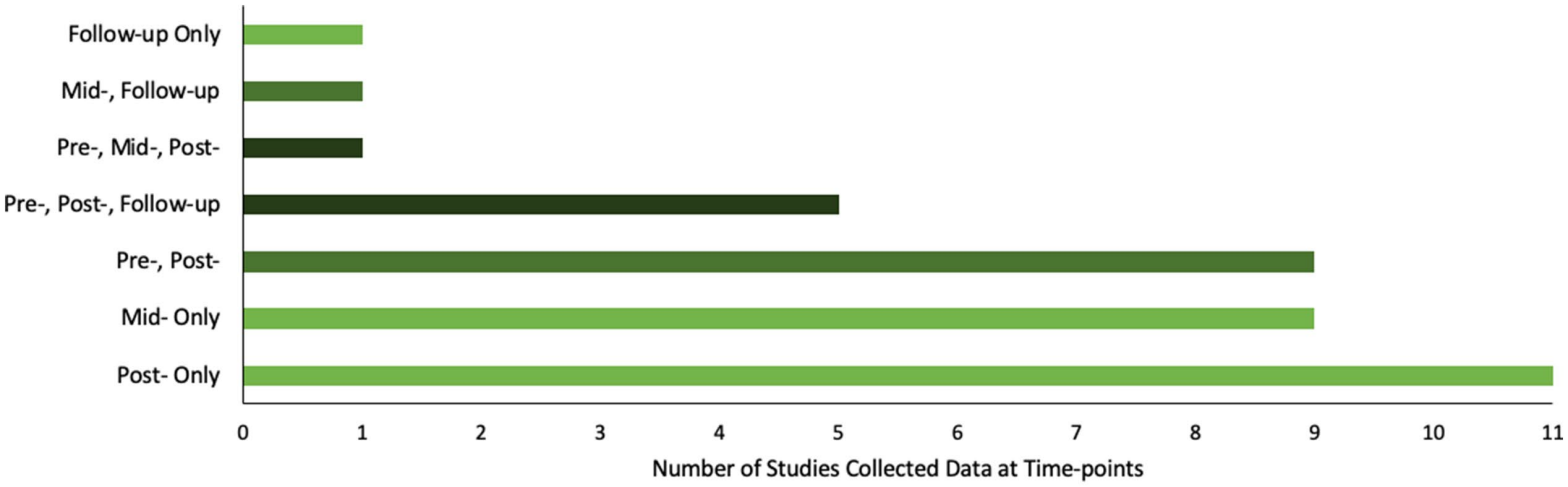
Data collection time-points.

Most studies used surveys (62%) and interviews (59%) to collect evaluation data, and many (43%) utilized psychometric tools. Most studies collected data at one time-point only (57%), either at mid-intervention (*n* = 9), post-intervention (*n* = 11) or follow-up (*n* = 1). Fifteen studies (27%) collected data at pre- and post-intervention, and five (14%) also collected data at follow-up (see Figure 4). Most (43%) follow-up measures were collected 2–3 months after the intervention, though one was collected at 12, and one at 24 months. Two longitudinal studies analyzed follow-up data, one at 10 years after a neighborhood regeneration project (Wilkie and Michialino, 2014), and one at 20 years in a cohort comparison study (Pillemer et al., 2010).

Most of the 18 studies using validated psychometric tools used 1–2 measures relating to mental wellbeing, except three that used 3–4 measures. Fourteen studies used psychometric tools reliably, taking pre- and post-measures, with four of these also collecting follow-up measures. These 14 studies largely applied within-group analysis, with three also applying between-group analysis where a comparison group was included. The remaining four studies used psychometric tools less reliably, opting for single time-point measurement for between-group comparison.

**Figure 5:**
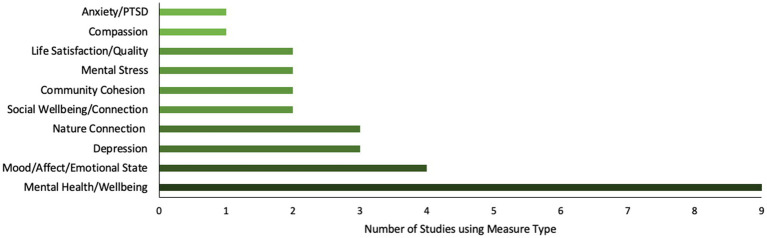
Types of outcome measures used.

The types of psychometric tools used are shown in Figure 5. Many used measures that assessed mental health or wellbeing, with the most used being the Warwick-Edinburgh Mental Wellbeing Scales (WEMWBS) and mental components from the 36-Item Short Form Survey (SF-36). Some studies used culturally specific scales (e.g., Chinese Happiness Inventory; Hsiao et al., 2020), and age-specific scales (e.g., Lubben Social Network Scale; Gagliardi et al., 2020).

### 3.5 Review question 4: Indications of perceived therapeutic mechanisms

**Figure 6:**
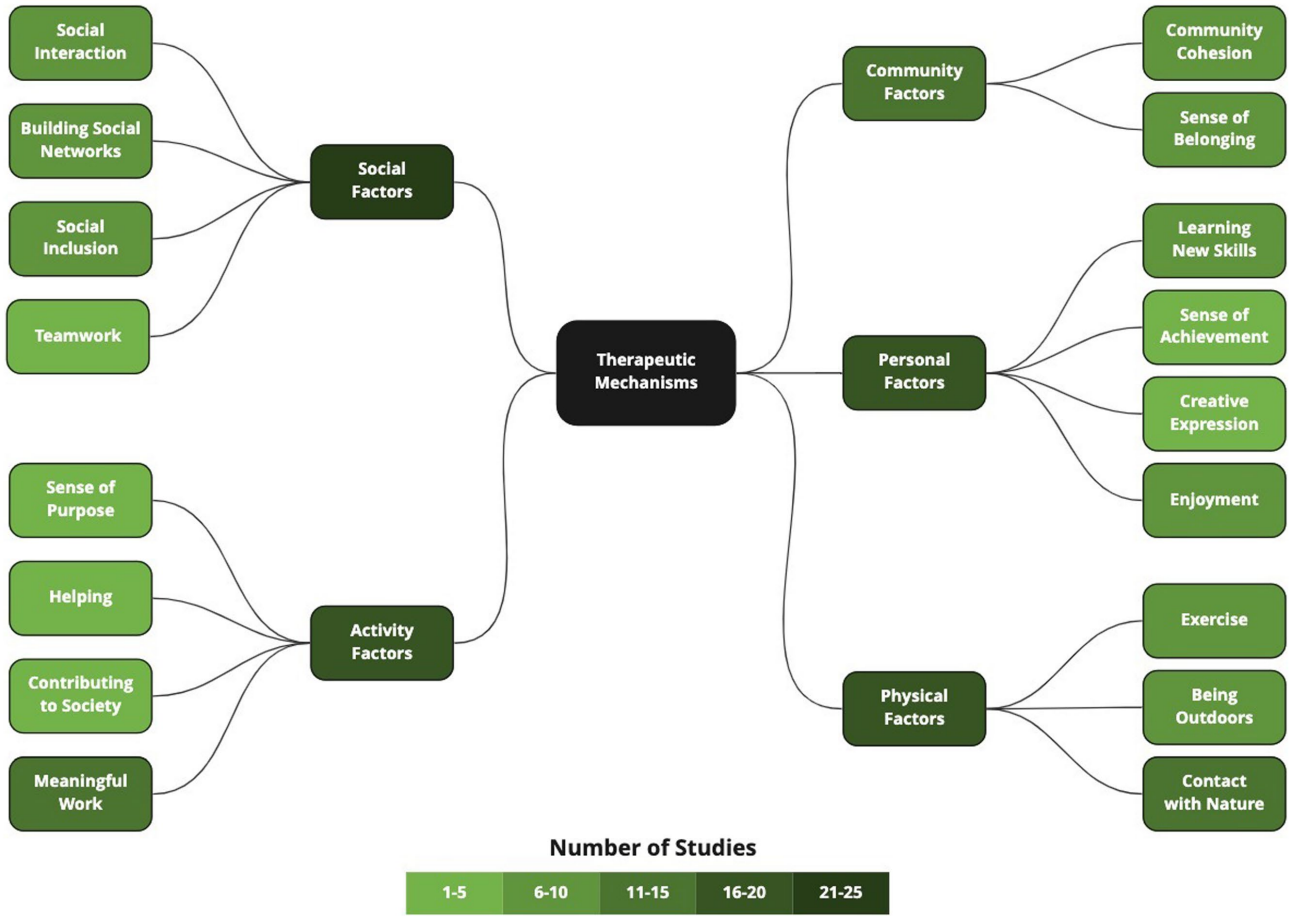
Perceived therapeutic mechanisms.

Analysis of the efficacy or effectiveness of the interventions was outside the scope of this review; however, it is worth noting that nearly all studies reported positive outcomes in either participant mental wellbeing, mood, or distress reduction. Discussion about the perceived or hypothesized therapeutic mechanisms was found in almost all (95%) of the studies (see Figure 6). Most studies (68%) reported social factors, including social interaction (24%), building social networks (24%), social inclusion (22%), and teamwork (14%). Factors relating to the nature of the activity were commonly reported (54% of studies), including participants feeling that the work was meaningful (38%), was helping others or the landscape (8%), was contributing to society (8%), and gave them a sense of purpose (8%).

Physical factors were commonly cited as therapeutically influential (54% of studies), including contact with nature (32%), being outdoors (27%), and exercise (19%). Personal factors were reported just as frequently (54% of studies), including learning new skills (22%), enjoyment (22%), as well as gaining self-efficacy and a sense of achievement (14%), and opportunity for creative expression (11%). Additionally, many studies (38%) discussed community factors, including community cohesion and connection (22%), and a sense of belonging (22%).

### 3.6 Review question 5: Indications of acceptability and challenges

Explicit indications of the acceptability of the interventions were found in only eight studies (22%), all of which cited challenges regarding the activities or the sustainability of programs. For example, the positive therapeutic impact relating to social factors was tempered by reports of interpersonal conflict within groups in four studies. Additionally, two studies reported problems with running activities in bad weather conditions, two reported time constraints as barriers to participation, and two mentioned sustainability issues due to funding or lack of leadership.

Four of these eight studies made positive references about acceptability, with two studies reporting that participants would continue the activities long-term, and one reporting that the intervention provided a “steppingstone” to further community engagement (Wilson et al., 2009).

### 3.7 Review question 6: Indications of eco-related distress

Given the relatively recent interest in researching eco-related distress, perhaps it is unsurprising that none of the studies explicitly measured or referenced eco-distress. However, nearly a third (30%) of the studies reported either participant concerns, views, or intended changes relating to the environment following the activities. For instance, one study reported that participants had expressed concerns about animals going extinct within local environments, which made them feel that longer-term volunteering commitment was needed despite the immediate gratification from the activity (Gooch, 2005). Two studies (O’Brien et al., 2011; Molsher and Townsend, 2016) reported that participants had gained awareness or understanding of issues in the environment and the need to conserve it from participation.

Six studies reported ecological attitude or behavior changes, largely through qualitative feedback. For instance, some participants reported that they had adopted pro-environmental behaviors or commitments following the intervention in three studies (e.g., “recycling,” Avon Wildlife Trust, 2021; “environmental activism,” Fraser et al., 2009). In the other three, some participants reported that they had adopted pro-environmental attitudes or beliefs (e.g., “developing environmental respect,” O’Brien et al., 2011).

## 4 Discussion

This novel and timely systematic scoping review has assessed current evidence for pro-ecological group-based community activities and their influence on mental wellbeing. Much like was found by Husk et al. (2016), studies reported a broad range of pro-ecological interventions, delivered in different ways over different durations, evaluated using varying study designs and assessment methods. Acceptability and challenges about the interventions were mentioned in several studies, and conclusions are mixed, given the considerable variation in activities studied and methodological quality. A full synthesis of the literature proved challenging, in part due to the decision to keep the scope broad to capture a diverse range of literature, including people across the lifespan and the globe.

Given this heterogeneity of the studies, and their variable quality and methods, synthesis and evaluation of wellbeing outcomes were neither planned nor attempted. Nonetheless, it is worth noting that key findings from almost all studies (see Table 1) reported positive outcomes in either participant mental wellbeing, mood, or distress reduction. It is reasonable to propose, then, that the common theoretical therapeutic mechanisms are more important than the discrete factors, duration, and location of the group-based pro-ecological activities.

Indeed, despite variations in activity types, samples, locations, and durations of participation, there are clear common findings and themes across studies which may relate to perceived therapeutic mechanisms, warranting further investigation and better experimental research designs. Social factors and perhaps distinguishing features of the pro-ecological activities appear to play an important role. Essentially, it seems that the activities bring people together socially, enabling them to work collaboratively on something that is meaningful and helpful to society at large. People thus talked about gaining a sense of belonging, purpose, and achievement. This fits with several theories including the Main Effect Model (Rook, 1990), which focuses on the rewarding nature of social integration through belonging and purpose, and the Warm-Glow Theory (Andreoni, 1989), which considers personal satisfaction and joy as arising from helping others or the environment. Where reduced distress was also reported, the Stress-Buffering Model (Cohen and Wills, 1985) could explain this through the stress coping mechanism believed to arise from social connection, and the Negative-state Relief Model (Cialdini and Kenrick, 1976), where pro-social actions may lead to improved mood.

Many activities were described as enjoyable, allowing people to learn alongside benefiting from exercise and being outdoors in nature. These findings are supported by existing theoretical approaches including the benefits nature can have upon stress (e.g., Stress Reduction Theory; Ulrich et al., 1991), and the sense of mastery and confidence arising from engaging in meaningful activity (e.g., Self-Efficacy Hypothesis; Craft, 2005). Moreover, longitudinal studies have implicated the importance of learning new skills for life satisfaction, self-esteem, and self-confidence (Feinstein and Hammond, 2004).

Given the blend of potential therapeutic mechanisms, and this review indicating broad improvement to mental wellbeing across ages and world locations, *“eco-caring together”* activities may offer something unique that requires further high-quality research. Many individual theories exist that attempt to explain the benefits of the isolated elements of the activities (e.g., exercise, pro-social behavior), but it is the compounding effect of these elements in a single activity that is intriguing, calling for synthesis and development of new theory. This scoping review provides a sound foundation and recommendation for further enquiry into the effectiveness and efficacy of such activities to promote mental health and wellbeing for a wide range of people across the world.

Just as Husk et al. (2016) recommended previously, more robust research is still needed. Randomized controlled designs could provide more valid evidence of the efficacy and effectiveness of such interventions. For instance, such trials could explore whether brief pro-ecological group-based interventions lead to substantive, sustained wellbeing improvements. If so, there may be the hope of such interventions being configured into non-clinical treatment programs that can be prescribed. Future research should examine what elements of the activities work for who, and the impact of individual characteristics on the benefits gained from participation. Comparisons between individual and collectivist societies would also be interesting.

An unforeseen finding was that some studies reported the activities increasing pro-ecological awareness (i.e., understanding, attitudinal, or behavioral changes) in some participants. This is promising, as it suggests that such activities could offer a way of engaging communities in direct, and ongoing indirect, benefits to ecological health. In the context of the ecological crisis, Empowerment Theory (Perkins and Zimmerman, 1995) posits that feelings of empowerment can enhance member participation and improvement of goal attainment. If this is the case, perhaps *“eco-caring together”* activities hold a self-motivating and self-sustaining potential for both personal and planetary health. However further research may also like to consider whether developing awareness of issues relating to planetary health could also lead to increased levels of eco-distress in some populations, and if so, explore wise and meaningful ways of incorporating this into new interventions.

### 4.1 Strengths and limitations

This scoping review was planned with the full protocol published prior to searching, making it transparent, clear, and open. A significant strength is its breadth, searching for sources across both published peer-reviewed and gray literature. For instance, reports from third-sector charities were found to meet eligibility criteria (e.g., Avon Wildlife Trust, 2021). This allowed this review to break from constraints to gain a fuller sense of what, where, why, and how *“eco-caring together”* is happening, and with who involved. This innovative review thus brings together diverse and varied literature, offering an integration of helpful theoretical approaches to understanding mental health, wellbeing, nature-based, group-focused, pro-ecological activities.

Such breadth and flexibility bring concurrent limitations. The inclusion of some non-academic sources (and thus the lack of peer-review) means caution had to be taken when interpreting findings, and the potential bias that could have arisen relating to funding ambitions. Dissertations were not included in the search strategy, and although this could have potentially led to the exclusion of relevant studies, the final included studies do still offer a comprehensive overview of the current state of the literature which has been subjected to the rigors of peer-review. The heterogeneity across samples, activities, and evaluation methods does however limit the degree to which an over-arching synthesis is possible, and thus conclusions about potential efficacy or effectiveness. All papers screened were in English meaning no papers were excluded on this criterion, which may have introduced publication bias.

We urgently need to find novel and scalable interventions to tackle the mental health crisis, and engaging people in activities that support planetary health offers an approach that can equally address the current ecological and climate crises. This scoping review paints a promising picture for pro-ecological group-based community interventions for mental health, wellbeing, and ecological health. A systematic approach to developing a stronger evidence base is encouraged, as well as a future systematic review of the efficacy and effectiveness of *“eco-caring together”* activities for mental wellbeing.

## Data availability statement

The original contributions presented in the study are included in the article/Supplementary material, further inquiries can be directed to the corresponding authors.

## Author contributions

KB: Conceptualization, Data curation, Formal analysis, Investigation, Methodology, Project administration, Validation, Visualization, Writing – original draft, Writing – review & editing. EM: Conceptualization, Formal analysis, Investigation, Methodology, Project administration, Supervision, Validation, Writing – review & editing. BC: Data curation, Formal analysis, Investigation, Validation, Writing – review & editing.

## Footnotes

^1^ https://osf.io/vmpr6
