# Supplementary material for: “Eco-caring together” pro-ecological group-based community interventions and mental wellbeing: a systematic scoping review
Source: Front Psychol. 2024 Apr 24;15:1288791. doi: 10.3389/fpsyg.2024.1288791 (PMC11076718; doi:10.3389/fpsyg.2024.1288791)
Supplement: Supplementary file 1 [file Data_Sheet_1.pdf]

Supplementary Material

"Eco-Caring Together" Pro-ecological group-based community interventions and mental wellbeing: A systematic scoping review

Appendix A: Search Terms

General Search Term

((("natur*" OR "environment*" OR "ecological" OR "green*" OR "park*" OR "forest" OR "wood*" OR "wild*" OR "beach" OR "river" OR "lake" OR "pond" OR "watershed") NEAR/2 ("regenerat*" OR "restor*" OR "preserv*" OR "enhanc*" OR "clean*" OR "creation")) OR ("conservation" OR "pro-ecological" OR "pro-environmental" OR "environmentally-friendly" OR "eco-friendly" OR "biodiversity" OR "rewild*" OR "re-green*" OR "climate change" OR "climate action" OR "purposeful" OR "litter picking" OR "tree planting")) AND ("group" OR "collectiv*" OR "commun*" OR "neighbourhood" OR "public") AND ("intervention" OR "programme" OR "social prescri*" OR "green prescri*" OR "initiative" OR "activi*" OR "volunt*" OR "help*" OR "steward*" OR "participati*") AND ("wellbeing" OR "well-being" OR "mental health" OR "mental illness" OR "distress" OR "stress" OR "self-esteem" OR "anxiety" OR "depression" OR "mood" OR "eco-distress" OR "eco-anxiety" OR "eco-depression" OR "benefits" OR "psychological" OR "life satisfaction" OR "happiness")

PsychNET Search Term

Keywords: ((("natur*" OR "environment*" OR "ecological" OR "green*" OR "park*" OR "forest" OR "wood*" OR "wild*" OR "beach" OR "river" OR "lake" OR "pond" OR "watershed") AND ("regenerat*" OR "restor*" OR "preserv*" OR "enhanc*" OR "clean*" OR "creation")) OR ("conservation" OR "pro-ecological" OR "pro-environmental" OR "environmentally-friendly" OR "eco-friendly" OR "biodiversity" OR "rewild*" OR "re-green*" OR "climate change" OR "climate action" OR "purposeful" OR "litter picking" OR "tree planting")) AND ("group" OR "collectiv*" OR "commun*" OR "neighbourhood" OR "public") AND ("intervention" OR "programme" OR "social prescri*" OR "green prescri*" OR "initiative" OR "activi*" OR "volunt*" OR "help*" OR "steward*" OR "participati*") AND ("wellbeing" OR "well-being" OR "mental health" OR "mental illness" OR "distress" OR "stress" OR "self-esteem" OR "anxiety" OR "depression" OR "mood" OR "eco-distress" OR "eco-anxiety" OR "eco-depression" OR "benefits" OR "psychological" OR "life satisfaction" OR "happiness")) OR Title: ((("natur*" OR "environment*" OR "ecological" OR "green*" OR "park*" OR "forest" OR "wood*" OR "wild*" OR "beach" OR "river" OR "lake" OR "pond" OR "watershed") AND ("regenerat*" OR "restor*" OR "preserv*" OR "enhanc*" OR "clean*" OR "creation")) OR ("conservation" OR "pro-ecological" OR "pro-environmental" OR "environmentally-friendly" OR "eco-friendly" OR "biodiversity" OR "rewild*" OR "re-green*" OR "climate change" OR "climate action" OR "purposeful" OR "litter picking" OR "tree planting")) AND ("group" OR "collectiv*" OR "commun*" OR "neighbourhood" OR "public") AND ("intervention" OR "programme" OR "social prescri*" OR "green prescri*" OR "initiative" OR "activi*" OR "volunt*" OR "help*" OR "steward*" OR "participati*") AND ("wellbeing" OR "well-being" OR "mental health" OR "mental illness" OR "distress" OR "stress" OR "self-esteem" OR "anxiety" OR "depression" OR "mood" OR "eco-distress" OR

“eco-anxiety” OR “eco-depression” OR “benefits” OR “psychological” OR “life satisfaction” OR “happiness”)) OR Abstract: (((“natur*” OR “environment*” OR “ecological” OR “green*” OR “park*” OR “forest” OR “wood*” OR “wild*” OR “beach” OR “river” OR “lake” OR “pond” OR “watershed”) AND (“regenerat*” OR “restor*” OR “preserv*” OR “enhanc*” OR “clean*” OR “creation”)) OR (“conservation” OR “pro-ecological” OR “pro-environmental” OR "environmentally-friendly" OR "eco-friendly" OR “biodiversity” OR “rewild*” OR “re-green*” OR “climate change” OR “climate action” OR “purposeful” OR “litter picking” OR “tree planting”)) AND (“group” OR “collectiv*” OR “commun*” OR “neighbourhood” OR “public”) AND (“intervention” OR “programme” OR “social prescri*” OR “green prescri*” OR “initiative” OR “activi*” OR “volunt*” OR “help*” OR “steward*” OR “participati*”) AND (“wellbeing” OR “well-being” OR “mental health” OR “mental illness” OR “distress” OR “stress” OR “self-esteem” OR “anxiety” OR “depression” OR “mood” OR “eco-distress” OR “eco-anxiety” OR “eco-depression” OR “benefits” OR “psychological” OR “life satisfaction” OR “happiness”)) AND Population Group: Human

PubMed Search Term

((((("natur*"[Text Word] OR "environment*"[Text Word] OR "ecological"[Text Word] OR "green*"[Text Word] OR "park*"[Text Word] OR "forest"[Text Word] OR "wood*"[Text Word] OR "wild*"[Text Word] OR "beach"[Text Word] OR "river"[Text Word] OR "lake"[Text Word] OR "pond"[Text Word] OR "watershed"[All Fields]) AND "n2"[All Fields]) AND ("regenerat*"[Text Word] OR "restor*"[Text Word] OR "preserv*"[Text Word] OR "enhanc*"[Text Word] OR "clean*"[Text Word] OR "creation"[Text Word])) OR ("conservation"[Text Word] OR "pro-ecological"[Text Word] OR "pro-environmental"[Text Word] OR "environmentally-friendly"[Text Word] OR "eco-friendly"[Text Word] OR "biodiversity"[Text Word] OR "rewild*"[Text Word] OR "re green*"[Text Word] OR "climate change"[Text Word] OR "climate action"[Text Word] OR "purposeful"[Text Word] OR "tree planting"[Text Word])) AND ("group"[Text Word] OR "collectiv*"[Text Word] OR "commun*"[Text Word] OR "neighbourhood"[Text Word] OR "public"[Text Word]) AND ("intervention"[Text Word] OR "programme"[Text Word] OR "social prescri*"[Text Word] OR "green prescri*"[Text Word] OR "initiative"[Text Word] OR "activi*"[Text Word] OR "volunt*"[Text Word] OR "help*"[Text Word] OR "steward*"[Text Word] OR "participati*"[Text Word]) AND ("wellbeing"[Text Word] OR "well-being"[Text Word] OR "mental health"[Text Word] OR "mental illness"[Text Word] OR "distress"[Text Word] OR "stress"[Text Word] OR "self-esteem"[Text Word] OR "anxiety"[Text Word] OR "depression"[Text Word] OR "mood"[Text Word] OR "eco-distress"[Text Word] OR "eco-anxiety"[Text Word] OR "eco-depression"[Text Word] OR "benefits"[Text Word] OR "psychological"[Text Word] OR "life satisfaction"[Text Word] OR "happiness"[Text Word])) AND (humans[Filter])

Scopus Search Term

TITLE-ABS-KEY (((("natur*" OR "environment*" OR "ecological" OR "green*" OR "park*" OR "forest" OR "wood*" OR "wild*" OR "beach" OR "river" OR "lake" OR "pond" OR "watershed") W/2 ("regenerat*" OR "restor*" OR "preserv*" OR "enhanc*" OR "clean*" OR "creation")) OR ("conservation" OR "pro-ecological" OR "pro-environmental" OR "environmentally-friendly" OR "eco-friendly" OR "biodiversity"

OR "rewild*" OR "re-green*" OR "climate change" OR "climate action" OR "purposeful" OR "litter picking" OR "tree planting")) AND ("group" OR "collectiv*" OR "commun*" OR "neighbourhood" OR "public") AND ("intervention" OR "programme" OR "social prescri*" OR "green prescri*" OR "initiative" OR "activi*" OR "volunt*" OR "help*" OR "steward*" OR "participati*") AND ("wellbeing" OR "well-being" OR "mental health" OR "mental illness" OR "distress" OR "stress" OR "self-esteem" OR "anxiety" OR "depression" OR "mood" OR "eco-distress" OR "eco-anxiety" OR "eco-depression" OR "benefits" OR "psychological" OR "life satisfaction" OR "happiness")) AND (LIMIT-TO (EXACTKEYWORD , "Human") OR LIMIT-TO (EXACTKEYWORD , "Humans")) AND (LIMIT-TO (DOCTYPE , "ar")) AND (LIMIT-TO (SUBJAREA , "MEDI") OR LIMIT-TO (SUBJAREA , "ENVI") OR LIMIT-TO (SUBJAREA , "SOCI") OR LIMIT-TO (SUBJAREA , "NURS") OR LIMIT-TO (SUBJAREA , "MULT") OR LIMIT-TO (SUBJAREA , "PSYC") OR LIMIT-TO (SUBJAREA , "HEAL") OR LIMIT-TO (SUBJAREA , "NEUR") OR LIMIT-TO (SUBJAREA , "EART"))

Web of Science Search Term

(((“natur*” OR “environment*” OR “ecological” OR “green*” OR “park*” OR “forest” OR “wood*” OR “wild*” OR “beach” OR “river” OR “lake” OR “pond” OR “watershed”) NEAR/2 (“regenerat*” OR “restor*” OR “preserv*” OR “enhanc*” OR “clean*” OR “creation”)) OR (“conservation” OR “pro-ecological” OR “pro-environmental” OR "environmentally-friendly" OR "eco-friendly" OR “biodiversity” OR “rewild*” OR “re-green*” OR “climate change” OR “climate action” OR “purposeful” OR “litter picking” OR “tree planting”)) AND (“group” OR “collectiv*” OR “commun*” OR “neighbourhood” OR “public”) AND (“intervention” OR “programme” OR “social prescri*” OR “green prescri*” OR “initiative” OR “activi*” OR “volunt*” OR “help*” OR “steward*” OR “participati*”) AND (“wellbeing” OR “well-being” OR “mental health” OR “mental illness” OR “distress” OR “stress” OR “self-esteem” OR “anxiety” OR “depression” OR “mood” OR “eco-distress” OR “eco-anxiety” OR “eco-depression” OR “benefits” OR “psychological” OR “life satisfaction” OR “happiness”) (Topic) and Humans (MeSH Headings) and Review Articles (Exclude – Document Types)

Appendix B: Excluded Sources (n=111)

Reasons for Exclusion

Article	Reason for exclusion
Anderson et al. (2021)	No mood, life satisfaction, mental health, affect or mental wellbeing assessment
Andow et al. (2016)	No mood, life satisfaction, mental health, affect or mental wellbeing assessment
Armsworth et al. (2013)	No mood, life satisfaction, mental health, affect or mental wellbeing assessment
Bawa et al. (2020)	No group working
Bazyk et al. (2009)	Not pro-ecological
Begum et al. (2021)	No mood, life satisfaction, mental health, affect or mental wellbeing assessment
Bellamy et al. (2017)	No group working
Bernard et al. (2022)	No mood, life satisfaction, mental health, affect or mental wellbeing assessment
Black et al. (2004)	Not pro-ecological
Breysse et al. (2015)	No group working
Brooks et al. (2010)	No mood, life satisfaction, mental health, affect or mental wellbeing assessment
Browder et al. (2002)	No mood, life satisfaction, mental health, affect or mental wellbeing assessment
Brown et al. (2011)	No mood, life satisfaction, mental health, affect or mental wellbeing assessment
Buijs et al. (2021)	No group working
Burgess et al. (2005	Exclusion reason: Review articles;
Burgess et al. (2009)	No mood, life satisfaction, mental health, affect or mental wellbeing assessment
Castell et al. (2020)	Exclusion reason: Review articles;
Chen et al. (2020)	No mood, life satisfaction, mental health, affect or mental wellbeing assessment
Clinch et al. (2021)	No mood, life satisfaction, mental health, affect or mental wellbeing assessment
Dallimer et al. (2014)	No group working
Dolrenry et al. (2016)	No mood, life satisfaction, mental health, affect or mental wellbeing assessment
Duvall et al. (2014)	Not pro-ecological
EcoHealthOntario et al. (2019)	No group working
El-Zein et al. (2006)	No group working
Elsey et al. (2014)	Exclusion reason: Research protocols;
ElZoghbi et al. (2014)	No group working
Gadd et al. (2005)	No mood, life satisfaction, mental health, affect or mental wellbeing assessment
Gao et al. (2018)	No mood, life satisfaction, mental health, affect or mental wellbeing assessment
Gibbs et al. (2013)	No group working
Gibson et al. (2021)	Not pro-ecological
Gilbertson et al. (2006)	No group working
Goffredo et al. (2010)	No mood, life satisfaction, mental health, affect or mental wellbeing assessment
Høegmark et al. (2021)	Not pro-ecological
Halpenny et al. (2003)	No mood, life satisfaction, mental health, affect or mental wellbeing assessment
Harper et al. (2006)	Not pro-ecological
Hartman et al. (2018)	No mood, life satisfaction, mental health, affect or mental wellbeing assessment
Havlick et al. (2021)	Not pro-ecological
Heinze et al. (2018)	No mood, life satisfaction, mental health, affect or mental wellbeing assessment
Hordyk et al. (2015)	No group working
Hughes et al. (2020)	No group working
Ihemezie et al. (2021)	Review or protocol
Janmaimool et al. (2016)	No group working
Januchowski-Hartley et al. (2012)	No mood, life satisfaction, mental health, affect or mental wellbeing assessment
Jiricka-Puerrer et al. (2019)	Not pro-ecological
Jones et al. (2018)	Experience via paid employment
Jones et al. (2020)	No mood, life satisfaction, mental health, affect or mental wellbeing assessment
Jung et al. (2017)	Not pro-ecological
Kaaya et al. (2017)	No mood, life satisfaction, mental health, affect or mental wellbeing assessment
Kafeety et al. (2020)	Not pro-ecological
Kaiser et al. (2011)	No mood, life satisfaction, mental health, affect or mental wellbeing assessment
Kalyanasundaram et al. (2021)	Review or protocol
Kamrowski et al. (2014)	No mood, life satisfaction, mental health, affect or mental wellbeing assessment
Kauneckis et al. (2009)	No mood, life satisfaction, mental health, affect or mental wellbeing assessment
Keane et al. (2016)	No mood, life satisfaction, mental health, affect or mental wellbeing assessment
Khadka et al. (2010)	No mood, life satisfaction, mental health, affect or mental wellbeing assessment
Kil et al. (2015)	Not pro-ecological
Kilpatrick et al. (2017)	Review or protocol

Kwok et al. (2017)	Experience via paid employment
Letcher et al. (2009)	Not pro-ecological
Li et al. (2017)	No mood, life satisfaction, mental health, affect or mental wellbeing assessment
Lirman et al. (2016)	No mood, life satisfaction, mental health, affect or mental wellbeing assessment
Liu et al. (2017)	No group working
Lumber et al. (2017)	No group working
Maraseni et al. (2014)	No mood, life satisfaction, mental health, affect or mental wellbeing assessment
Marselle et al. (2015)	Not pro-ecological
Martin et al. (2008)	No mood, life satisfaction, mental health, affect or mental wellbeing assessment
Maund et al. (2019)	Not pro-ecological
Mehta et al. (2001)	No mood, life satisfaction, mental health, affect or mental wellbeing assessment
Mor et al. (2018)	No mood, life satisfaction, mental health, affect or mental wellbeing assessment
Morón et al. (2006)	Review or protocol
Naeem et al. (2016)	Review or protocol
Nothwehr et al. (2019)	No mood, life satisfaction, mental health, affect or mental wellbeing assessment
OduorAMO et al. (2020)	Experience via paid employment
Oh et al. (2021)	No group working
Patrick et al. (2011)	No group working
Pearson et al. (2020)	Review or protocol
Pedersen et al. (2016)	No mood, life satisfaction, mental health, affect or mental wellbeing assessment
Pfefferbaum et al. (2013)	No mood, life satisfaction, mental health, affect or mental wellbeing assessment
Pich et al. (2020)	Review or protocol
Pillemer et al. (2017)	No mood, life satisfaction, mental health, affect or mental wellbeing assessment
Pradyumna et al. (2020)	No mood, life satisfaction, mental health, affect or mental wellbeing assessment
Pryor et al. (2006)	Not pro-ecological
Reese et al. (2020)	No group working
Richards et al. (2020)	No group working
Riehl et al. (2015)	No mood, life satisfaction, mental health, affect or mental wellbeing assessment
Rogers et al. (2021)	No group working
Rostami et al. (2014)	Not pro-ecological
Schram-Bijkerk et al. (2018)	Review or protocol
Silva et al. (2005)	No group working
Soga et al. (2016)	No group working
Sotomayor et al. (2014)	No mood, life satisfaction, mental health, affect or mental wellbeing assessment
Spiteri et al. (2008)	No mood, life satisfaction, mental health, affect or mental wellbeing assessment
Stegeman et al. (2020)	Review or protocol
Suarez et al. (2018)	No group working
Timler et al. (2020)	No group working
Townsend et al. (2005)	Not pro-ecological
Ullah et al. (2021)	No mood, life satisfaction, mental health, affect or mental wellbeing assessment
Wai et al. (2012)	No group working
Wallace et al. (2008)	No group working
Wang et al. (2021)	No mood, life satisfaction, mental health, affect or mental wellbeing assessment
Ward et al. (2018)	No mood, life satisfaction, mental health, affect or mental wellbeing assessment
Wheeler et al. (2015)	No group working
White et al. (2018)	No mood, life satisfaction, mental health, affect or mental wellbeing assessment
White et al. (2020)	No group working
Wickrama et al. (2011)	No group working
Woodgate et al. (2015)	No mood, life satisfaction, mental health, affect or mental wellbeing assessment
Yessoufou et al. (2020)	Not pro-ecological
Young et al. (2020)	Not pro-ecological
Zhang et al. (2021)	Review or protocol
Zhao et al. (2019)	No group working
Zhao et al. (2021)	No mood, life satisfaction, mental health, affect or mental wellbeing assessment

References for Excluded Studies

Anderson, V., Gough, W. A., & Agic, B. (2021). Nature-based equity: An assessment of the public health impacts of green infrastructure in Ontario Canada. *International Journal of Environmental Research and Public Health, 18*(11). doi:10.3390/ijerph18115763

Andow, D. A., Borgida, E., Hurley, T. M., & Williams, A. L. (2016). Recruitment and retention of volunteers in a citizen science network to detect invasive species on private lands. *Environmental Management, 58*(4), 606-618. doi:10.1007/s00267-016-0746-7

Armsworth, P. R., Cantú-Salazar, L., Parnell, M., Booth, J. E., Stoneman, R., & Davies, Z. G. (2013). Opportunities for cost-sharing in conservation: Variation in volunteering effort across protected areas. *PLOS ONE, 8*(1). doi:10.1371/journal.pone.0055395

Bawa, K. S., Nawn, N., Chellam, R., Krishnaswamy, J., Mathur, V., Olsson, S. B., . . . Quader, S. (2020). Envisioning a biodiversity science for sustaining human well-being. *Proceedings of the National Academy of Sciences, 117*(42), 25951-25955. doi:10.1073/pnas.2018436117

Bazyk, S., & Bazyk, J. (2009). Meaning of occupation-based groups for low-income urban youths attending after-school care. *American Journal of Occupational Therapy, 63*(1), 69-80. doi:10.5014/ajot.63.1.69

Begum, F., Lobry de Bruyn, L., Kristiansen, P., & Islam, M. A. (2021). Institutionalising co-management activities for conservation of forest resources: Evidence from the Sundarban mangrove forest management of Bangladesh. *Journal of Environmental Management, 298*, 113504. doi:10.1016/j.jenvman.2021.113504

Bellamy, C. C., van der Jagt, A. P. N., Barbour, S., Smith, M., & Moseley, D. (2017). A spatial framework for targeting urban planning for pollinators and people with local stakeholders: A route to healthy, blossoming communities? *Environmental Research, 158*, 255-268. doi:10.1016/j.envres.2017.06.023

Bernard, M. L. (2022). Community engagement and perceptions in marine conservation in the Caribbean. (83). *ProQuest Information & Learning, US*.

Black, W., & Living, R. (2004). Volunteerism as an occupation and its relationship to health and wellbeing. *British Journal of Occupational Therapy, 67*(12), 526-532. doi:10.1177/030802260406701202

Breysse, J., Dixon, S. L., Jacobs, D. E., Lopez, J., & Weber, W. (2015). Self-reported health outcomes associated with green-renovated public housing among primarily elderly residents. *Journal of Public Health Management and Practice, 21*(4), 355-367. doi:10.1097/PHH.0000000000000199

Brooks, J. S. (2010). Economic and social dimensions of environmental behavior: Balancing conservation and development in Bhutan. *Conservation Biology, 24*(6), 1499-1509. doi:10.1111/j.1523-1739.2010.01512.x

Browder, J. O. (2002). Conservation and development projects in the Brazilian Amazon: Lessons from the community initiative program in Rondônia. *Environmental Management, 29*(6), 750-762. doi:10.1007/s00267-001-2613-3

Brown, D. R., Dettmann, P., Rinaudo, T., Tefera, H., & Tofu, A. (2011). Poverty alleviation and environmental restoration using the clean development mechanism: A case study from Humbo, Ethiopia. *Environmental Management, 48*(2), 322-333. doi:10.1007/s00267-010-9590-3

Buijs, A., & Jacobs, M. (2021). Avoiding negativity bias: Towards a positive psychology of human-wildlife relationships. *Ambio, 50*(2), 281-288. doi:10.1007/s13280-020-01394-w

Burgess, C. P., Johnston, F. H., Berry, H. L., McDonnell, J., Yibarbuk, D., Gunabarra, C., . . . Bailie, R. S. (2009). Healthy country, healthy people: The relationship between Indigenous health status and "caring for country". *Medical Journal of Australia, 190*(10), 567-572. doi:10.5694/j.1326-5377.2009.tb02566.x

Burgess, C. P., Johnston, F. H., Bowman, D. M., & Whitehead, P. J. (2005). Healthy country: Healthy people? Exploring the health benefits of indigenous natural resource management. *Australian and New Zealand Journal of Public Health, 29*(2), 117-122. doi:10.1111/j.1467-842x.2005.tb00060.x

Castell, C. (2020). Nature and health: A necessary alliance. *Gaceta Sanitara, 34*(2), 194-196. doi:10.1016/j.gaceta.2019.05.016

Chen, J.-C., Chang, Q.-X., Liang, C.-C., Hsieh, J.-G., Liu, P. P.-S., Yen, C.-F., & Loh, C.-H. (2020). Potential benefits of environmental volunteering programs of the health of older adults: A pilot study. *Archives of Gerontology and Geriatrics, 90*. doi:10.1016/j.archger.2020.104113

Clinch, M. (2021). Environmental stewardship in austere times: Nurturing sustainable socio-ecological relations. *Critical Public Health, 31*(3), 245-254. doi:10.1080/09581596.2020.1853057

Dallimer, M., Davies, Z. G., Irvine, K. N., Maltby, L., Warren, P. H., Gaston, K. J., & Armsworth, P. R. (2014). What personal and environmental factors determine frequency of urban greenspace use? *International Journal of Environmental Research and Public Health, 11*(8), 7977-7992. doi:10.3390/ijerph110807977

Dolrenry, S., Hazzah, L., & Frank, L. G. (2016). Conservation and monitoring of a persecuted African lion population by Maasai warriors. *Conservation Biology, 30*(3), 467-475. doi:10.1111/cobi.12703

Duvall, J., & Kaplan, R. (2014). Enhancing the well-being of veterans using extended group-based nature recreation experiences. *Journal of Rehabilitation Research and Development, 51*(5), 685-696. doi:10.1682/JRRD.2013.08.0190

EcoHlth, O., & Kingsley, M. (2019). Climate change, health and green space co-benefits. *Health promotion and chronic disease prevention in Canada: research, policy and practice, 39*(4), 131-135. doi:10.24095/hpcdp.39.4.04

El Zoghbi, M. B., & El Ansari, W. (2014). University students as recipients of and contributors to information on climate change: Insights from South Africa and implications for well-being. *Central European Journal of Public Health, 22*(2), 125-132. doi:10.21101/cejph.a3999

El-Zein, A., Nasrallah, R., & Nuwayhid, I. (2006). Determinants of the willingness-to-participate in an environmental intervention in a Beirut neighborhood. *Environmental Management, 37*(2), 200-208. doi:10.1007/s00267-004-0375-4

Elsey, H., Bragg, R., Elings, M., Cade, J. E., Brennan, C., Farragher, T., . . . Murray, J. (2014). Understanding the impacts of care farms on health and well-being of disadvantaged populations: A protocol of the Evaluating Community Orders (ECO) pilot study. *BMJ Open, 4*(10). doi:10.1136/bmjopen-2014-0065361

Gadd, M. E. (2005). Conservation outside of parks: Attitudes of local people in Laikipia, Kenya. *Environmental Conservation, 32*(1), 50-63. doi:10.1017/S0376892905001918

Gao, Y., Church, S. P., Peel, S., & Prokopy, L. S. (2018). Public perception towards river and water conservation practices: Opportunities for implementing urban stormwater management practices. *Journal of Environmental Management, 223*, 478-488. doi:10.1016/j.jenvman.2018.06.059

Gibbs, L., Waters, E., Bryant, R. A., Pattison, P., Lusher, D., Harms, L., . . . Forbes, D. (2013). Beyond bushfires: Community, resilience and recovery – A longitudinal mixed method study of the

medium to long term impacts of bushfires on mental health and social connectedness. *BMC Public Health, 13*. doi:10.1186/1471-2458-13-1036

Gibson, K., Little, J., Cowlishaw, S., Ipitoa Toromon, T., Forbes, D., & O'Donnell, M. (2021). Piloting a scalable, post-trauma psychosocial intervention in Tuvalu: The Skills for Life Adjustment and Resilience (SOLAR) program. *European Journal of Psychotraumatology, 12*(1), 1948253. doi:10.1080/20008198.2021.1948253

Gilbertson, J., Stevens, M., Stiell, B., & Thorogood, N. (2006). Home is where the hearth is: Grant recipients' views of England's home energy efficiency scheme (Warm Front). *Social Science & Medicine, 63*(4), 946-956. doi:10.1016/j.socscimed.2006.02.021

Goffredo, S., Pensa, F., Neri, P., Orlandi, A., Gagliardi, M. S., Velardi, A., . . . Zaccanti, F. (2010). Unite research with what citizens do for fun: "Recreational monitoring" of marine biodiversity. *Ecological Applications, 20*(8), 2170-2187. doi:10.1890/09-1546.1

Halpenny, E. A., & Caissie, L. T. (2003). Volunteering on nature conservation projects: Volunteer experience, attitudes and values. *Tourism Recreation Research, 28*(3), 25-33. doi: 10.1080/02508281.2003.11081414

Harper, N., & Scott, D. G. (2006). Therapeutic outfitting: Enhancing conventional adolescent mental health interventions through innovative collaborations with a wilderness experience programme. *Therapeutic Communities, 27*(4), 549-571.

Hartman, B. D., & Cleveland, D. A. (2018). The socioeconomic factors that facilitate or constrain restoration management: Watershed rehabilitation and wet meadow (bofedal) restoration in the Bolivian Andes. *Journal of Environmental Management, 209*, 93-104. doi:10.1016/j.jenvman.2017.12.025

Havlick, D. G., Cerveny, L. K., & Derrien, M. M. (2021). Therapeutic landscapes, outdoor programs for veterans, and public lands. *Social Science & Medicine, 268*. doi:10.1016/j.socscimed.2020.113540

Heinze, J. E., Krusky-Morey, A., Vagi, K. J., Reischl, T. M., Franzen, S., Pruett, N. K., . . . Zimmerman, M. A. (2018). Busy streets theory: The effects of community-engaged greening on violence. *American Journal of Community Psychology, 62*(1-2), 101-109. doi:10.1002/ajcp.12270

Høegmark, S., Andersen, T. E., Grahn, P., Mejldal, A., & Roessler, K. K. (2021). The Wildman programme—Rehabilitation and reconnection with nature for men with mental or physical health problems—A matched-control study. *International Journal of Environmental Research and Public Health, 18*(21). doi:10.3390/ijerph182111465

Hordyk, S. R., Hanley, J., & Richard, E. (2015). "Nature is there; its free": Urban greenspace and the social determinants of health of immigrant families. *Health & Place, 34*, 74-82. doi:10.1016/j.healthplace.2015.03.016

Hughes, J., De Ruyck, C., Emmens, T., Bradbury, R. B., & Jefferson, R. (2020). In a mental-health care setting, can nature conservation and health priorities align? *Journal of Interprofessional Care, 34*(1), 97-106. doi:10.1080/13561820.2019.1621276

Ihemezie, E. J., Nawrath, M., Strauß, L., Stringer, L. C., & Dallimer, M. (2021). The influence of human values on attitudes and behaviours towards forest conservation. *Journal of Environmental Management, 292*, 112857. doi:10.1016/j.jenvman.2021.112857

Janmaimool, P., & Denpaiboon, C. (2016). Evaluating determinants of rural villagers' engagement in conservation and waste management behaviors based on integrated conceptual framework of pro-environmental behavior. *Life Sciences, Society and Policy, 12*(1), 12. doi:10.1186/s40504-016-0045-3

Januchowski-Hartley, S. R., Moon, K., Stoeckl, N., & Gray, S. (2012). Social factors and private benefits influence landholders' riverine restoration priorities in tropical Australia. *Journal of Environmental Management, 110*, 20-26. doi:10.1016/j.jenvman.2012.05.011

Jiricka-Puerrer, A., Tadini, V., Salak, B., Taczanowska, K., Tucki, A., & Senes, G. (2019). Do protected areas contribute to health and well-being? A cross-cultural comparison. *International Journal of Environmental Research and Public Health, 16*(7). doi:10.3390/ijerph16071172

Jones, M. S., & Niemiec, R. M. (2020). Social-psychological correlates of personal-sphere and diffusion behavior for wildscape gardening. *Journal of Environmental Management, 276*, 111271. doi:10.1016/j.jenvman.2020.111271

Jones, R., Thurber, K. A., Wright, A., Chapman, J., Donohoe, P., Davis, V., & Lovett, R. (2018). Associations between participation in a ranger program and health and wellbeing outcomes among aboriginal and torres strait islander people in central Australia: A proof of concept study. *International Journal of Environmental Research and Public Health, 15*(7). doi:10.3390/ijerph15071478

Jung, M., Jonides, J., Northouse, L., Berman, M. G., Koelling, T. M., & Pressler, S. J. (2017). Randomized crossover study of the natural restorative environment intervention to improve attention and mood in heart failure. *Journal of Cardiovascular Nursing, 32*(5), 464-479. doi:10.1097/JCN.0000000000000368

Kaaya, E., & Chapman, M. (2017). Micro-credit and community wildlife management: complementary strategies to improve conservation outcomes in Serengeti National Park, Tanzania. *Environmental Management, 60*(3), 464-475. doi:10.1007/s00267-017-0856-x

Kafeety, A., Henderson, S. B., Lubik, A., Kancir, J., Kosatsky, T., & Schwandt, M. (2020). Social connection as a public health adaptation to extreme heat events. *Canadian Journal of Public Health, 111*(6), 876-879. doi:10.17269/s41997-020-00309-2

Kaiser, F. G., & Byrka, K. (2011). Environmentalism as a trait: Gauging people's prosocial personality in terms of environmental engagement. *International Journal of Psychology, 46*(1), 71-79. doi:10.1080/00207594.2010.516830

Kalyanasundaram, M., Sabde, Y., Annerstedt, K. S., Singh, S., Sahoo, K. C., Parashar, V., . . . Diwan, V. (2021). Effects of improved information and volunteer support on segregation of solid waste at the household level in urban settings in Madhya Pradesh, India (I-MISS): Protocol of a cluster randomized controlled trial. *BMC Public Health, 21*(1), 694. doi:10.1186/s12889-021-10693-0

Kamrowski, R. L., Sutton, S. G., Tobin, R. C., & Hamann, M. (2014). Potential applicability of persuasive communication to light-glow reduction efforts: A case study of marine turtle conservation. *Environmental Management, 54*(3), 583-595. doi:10.1007/s00267-014-0308-9

Kauneckis, D., & York, A. M. (2009). An empirical evaluation of private landowner participation in voluntary forest conservation programs. *Environmental Management, 44*(3), 468-484. doi:10.1007/s00267-009-9327-3

Keane, A., Gurd, H., Kaelo, D., Said, M. Y., Leeuw, J. D., Rowcliffe, J. M., & Homewood, K. (2016). Gender differentiated preferences for a community-based conservation initiative. *PLOS ONE, 11*(3). doi:10.1371/journal.pone.0152432

Khadka, D., & Nepal, S. K. (2010). Local responses to participatory conservation in Annapurna conservation area, Nepal. *Environmental Management, 45*(2), 351-362. doi:10.1007/s00267-009-9405-6

Kil, N., Holland, S. M., & Stein, T. V. (2015). Experiential benefits, place meanings, and environmental setting preferences between proximate and distant visitors to a national scenic trail. *Environmental Management, 55*(5), 1109-1123. doi:10.1007/s00267-015-0445-9

Kilpatrick, A. M., Salkeld, D. J., Titcomb, G., & Hahn, M. B. (2017). Conservation of biodiversity as a strategy for improving human health and well-being. *Philosophical Transactions of the Royal Society B, 372*(1722). doi:10.1098/rstb.2016.0131

Kwok, R. K., McGrath, J. A., Lowe, S. R., Engel, L. S., Jackson, W. B., II, Curry, M. D., . . . Sandler, D. P. (2017). Mental health indicators associated with oil spill response and clean-up: Cross-sectional analysis of the GULF study cohort. *The Lancet Public Health, 2*(12), E560-E567. doi:10.1016/S2468-2667(17)30194-9

Letcher, A. S., & Perlow, K. M. (2009). Community-based participatory research shows how a community initiative creates networks to improve well-being. *American Journal of Preventive Medicine, 37*(6 Suppl 1), S292-299. doi:10.1016/j.amepre.2009.08.008

Li, H., Bennett, M. T., Jiang, X., Zhang, K., & Yang, X. (2017). Rural household preferences for active participation in "payment for ecosystem service" programs: A case in the miyun reservoir catchment, China. *PLOS ONE, 12*(1). doi:10.1371/journal.pone.0169483

Lirman, D., & Schopmeyer, S. (2016). Ecological solutions to reef degradation: Optimizing coral reef restoration in the Caribbean and Western Atlantic. *PeerJ,* (10). doi:10.7717/peerj.2597

Liu, H., & Li, F. (2017). The relationships between urban parks, residents' physical activity, and mental health benefits: A case study from Beijing, China. *Journal of Environmental Management, 190*, 223-230. doi:10.1016/j.jenvman.2016.12.058

Lumber, R., Richardson, M., & Sheffield, D. (2017). Beyond knowing nature: Contact, emotion, compassion, meaning, and beauty are pathways to nature connection. *PLOS ONE, 12*(5), e0177186. doi:10.1371/journal.pone.0177186

Maraseni, T. N., Neupane, P. R., Lopez-Casero, E., & Cadman, T. (2014). An assessment of the impacts of the REDD plus pilot project on community forests user groups (CFUGs) and their community forests in Nepal. *Journal of Environmental Management, 136*, 37-46. doi:10.1016/j.jenvman.2014.01.011

Marselle, M. R., Irvine, K. N., Lorenzo-Arribas, A., & Warber, S. L. (2015). Moving beyond green: Exploring the relationship of environment type and indicators of perceived environmental quality on emotional well-being following group walks. *International Journal of Environmental Research and Public Health, 12*(1), 106-130. doi:10.3390/ijerph120100106

Martin, L., Miranda, B., & Bean, M. (2008). An exploration of spousal separation and adaptation to long-term disability: Six elderly couples engaged in a horticultural programme. *Occupational Therapy International, 15*(1), 45-55. doi:10.1002/oti.240

Maund, P. R., Irvine, K. N., Reeves, J., Strong, E., Cromie, R., Dallimer, M., & Davies, Z. G. (2019). Wetlands for wellbeing: Piloting a nature-based health intervention for the management of anxiety and depression. *International Journal of Environmental Research and Public Health, 16*(22). doi:10.3390/ijerph16224413

Mehta, J. N., & Heinen, J. T. (2001). Does community-based conservation shape favorable attitudes among locals? An empirical study from Nepal. *Environmental Management, 28*(2), 165-177. doi:10.1007/s002670010215

Mor, S. M., Norris, J. M., Bosward, K. L., Toribio, J. A. L. M. L., Ward, M. P., Gongora, J., . . . Zaki, S. (2018). One health in our backyard: Design and evaluation of an experiential learning experience for veterinary medical students. *One Health, 5*, 57-64. doi:10.1016/j.onehlt.2018.05.001

Morón, C. (2006). Food-based nutrition interventions at community level. *British Journal of Nutrition, 96* (S1), S20-22. doi:10.1079/bjn20061693

Naeem, S., Chazdon, R., Duffy, J. E., Prager, C., & Worm, B. (2016). Biodiversity and human well-being: An essential link for sustainable development. *Proceedings of The Royal Society B, 283*(1844). doi:10.1098/rspb.2016.2091

Nothwehr, F., & Rohlman, D. (2019). Employer-supported volunteerism in rural worksites. *Workplace Health & Safety, 67*(10), 512-519. doi:10.1177/2165079919862295

Oduor, A. M. O. (2020). Livelihood impacts and governance processes of community-based wildlife conservation in Maasai Mara ecosystem, Kenya. *Journal of Environmental Management, 260*. doi:10.1016/j.jenvman.2020.110133

Oh, R. R. Y., Fielding, K. S. S., Chang, C.-C., Nghiem, L. T. P., Tan, C. L. Y., Quazi, S. A., . . . Fuller, R. A. (2021). Health and wellbeing benefits from nature experiences in tropical settings depend on strength of connection to nature. *International Journal of Environmental Research and Public Health, 18*(19). doi:10.3390/ijerph181910149

Patrick, R., & Capetola, T. (2011). It's here! Are we ready? Five case studies of health promotion practices that address climate change from within Victorian health care settings. *Health Promotion Journal of Australia, 22*, S61-S67. doi:10.1071/HE11461

Pearson, A. L., Pfeiffer, K. A., Gardiner, J., Horton, T., Buxton, R. T., Hunter, R. F., . . . McDade, T. (2020). Study of active neighborhoods in Detroit (StAND): Study protocol for a natural experiment evaluating the health benefits of ecological restoration of parks. *BMC Public Health, 20*(1). doi:10.1186/s12889-020-08716-3

Pedersen, I., Dalskau, L. H., Ihlebaek, C., & Patil, G. (2016). Content and key components of vocational rehabilitation on care farms for unemployed people with mental health problems: A case study report. *Work, 53*(1), 21-30. doi:10.3233/WOR-152212

Pfefferbaum, R. L., Pfefferbaum, B., Van Horn, R. L., Neas, B. R., & Houston, J. B. (2013). Building community resilience to disasters through a community-based intervention: CART applications. *Journal of Emergency Management, 11*(2), 151-159. doi:10.5055/jem.2013.0134

Pich, J. (2020). Participation in environmental enhancement and conservation activities for health and well-being in adults: A review of quantitative and qualitative evidence. *Public Health Nursing, 37*(1), 144-146. doi:10.1111/phn.12680

Pillemer, K., Wells, N. M., Meador, R. H., Schultz, L., Henderson, C. R., & Cope, M. T. (2017). Engaging older adults in environmental volunteerism: The retirees in service to the environment program. *The Gerontologist, 57*(2), 367-375.

Pradyumna, A., Mishra, A., Utzinger, J., & Winkler, M. S. (2020). Perceived health impacts of watershed development projects in Southern India: A qualitative study. *International Journal of Environmental Research and Public Health, 17*(10). doi:10.3390/ijerph17103448

Pryor, A., Townsend, M., Maller, C., & Field, K. (2006). Health and well-being naturally: 'Contact with nature' in health promotion for targeted individuals, communities and populations. *Health Promotion Journal of Australia, 17*(2), 114-123. doi:10.1071/he06114

Reese, R. F., Seitz, C. M., Gosling, M., & Craig, H. (2020). Using photovoice to foster a student vision for natural spaces on a college campus in the Pacific Northwest United States. *International Journal of Environmental Health Research, 30*(3), 296-311. doi:10.1080/09603123.2019.1593950

Richards, D. R., Fung, T. K., Leong, R. A. T., Sachidhanandam, U., Drillet, Z., & Edwards, P. J. (2020). Demographic biases in engagement with nature in a tropical Asian city. *PLOS ONE, 15*(4), e0231576. doi:10.1371/journal.pone.0231576

Riehl, B., Zerriffi, H., & Naidoo, R. (2015). Effects of community-based natural resource management on household welfare in Namibia. *PLOS ONE, 10*(5). doi:10.1371/journal.pone.0125531

Rogers, H. H. (2021). A daily practice for planetary health. *Creative Nursing, 27*(4), 267-268. doi:10.1891/cn-2021-0025

Rostami, R., Lamit, H., Khoshnava, S. M., & Rostami, R. (2014). The role of historical persian gardens on the health status of contemporary urban residents' gardens and health status of contemporary urban residents. *EcoHealth, 11*(3), 308-321. doi:10.1007/s10393-014-0939-6

Schram-Bijkerk, D., Otte, P., Dirven, L., & Breure, A. M. (2018). Indicators to support healthy urban gardening in urban management. *Science of the Total Environment, 621*, 863-871. doi:10.1016/j.scitotenv.2017.11.160

Silva, H. P., Boscolo, O. H., Nascimento, G., Obermüller, F., & Strelow, F. (2005). Biodiversity conservation and human well-being: Challenges for the populations and protected areas of the Brazilian Atlantic Forest. *EcoHealth, 2*(4), 333-342. doi:10.1007/s10393-005-8361-8

Soga, M., Gaston, K. J., Yamaura, Y., Kurisu, K., & Hanaki, K. (2016). Both direct and vicarious experiences of nature affect children's willingness to conserve biodiversity. *International Journal of Environmental Research and Public Health, 13*(6). doi:10.3390/ijerph13060529

Sotomayor, S., Barbieri, C., Stanis, S. W., Aguilar, F. X., & Smith, J. W. (2014). Motivations for recreating on farmlands, private forests, and state or national parks. *Environmental Management, 54*(1), 138-150. doi:10.1007/s00267-014-0280-4

Spiteri, A., & Nepal, S. K. (2008). Evaluating local benefits from conservation in Nepal's Annapurna Conservation Area. *Environmental Management, 42*(3), 391-401. doi:10.1007/s00267-008-9130-6

Stegeman, I., Godfrey, A., Romeo-Velilla, M., Bell, R., Staatsen, B., van der Vliet, N., . . . Costongs, C. (2020). Encouraging and enabling lifestyles and behaviours to simultaneously promote environmental sustainability, health and equity: Key policy messages from INHERIT. *International Journal of Environmental Research and Public Health, 17*(19). doi:10.3390/ijerph17197166

Suarez, A., Arias-Arévalo, P., Martinez-Mera, E., Granobles-Torres, J. C., & Enríquez-Acevedo, T. (2018). Involving victim population in environmentally sustainable strategies: An analysis for post-conflict Colombia. *Science of The Total Environment, 643*, 1223-1231. doi:10.1016/j.scitotenv.2018.06.262

Timler, K., & Sandy, D. W. (2020). Gardening in ashes: The possibilities and limitations of gardening to support indigenous health and well-being in the context of wildfires and colonialism. *International Journal of Environmental Research and Public Health, 17*(9). doi:10.3390/ijerph17093273

Townsend, M. (2005). Health, wellbeing and social capital benefits of open space use within the City of Knox. *Deakin Research Online.*

Ullah, A., Sam, A. S., Sathyan, A. R., Mahmood, N., Zeb, A., & Kächele, H. (2021). Role of local communities in forest landscape restoration: Key lessons from the Billion Trees Afforestation Project, Pakistan. *Science of The Total Environment, 772*. doi:10.1016/j.scitotenv.2021.145613

Wai, K. T., Htun, P. T., Oo, T., Myint, H., Lin, Z., Kroeger, A., . . . Petzold, M. (2012). Community-centred eco-bio-social approach to control dengue vectors: An intervention study from Myanmar. *Pathogens and Global Health, 106*(8), 461-468. doi:10.1179/2047773212Y.0000000057

Wallace, G. N., Theobald, D. M., Ernst, T., & King, K. (2008). Assessing the ecological and social benefits of private land conservation in Colorado. *Conservation Biology, 22*(2), 284-296. doi:10.1111/j.1523-1739.2008.00895.x

Wang, H., & Mangmeechai, A. (2021). Understanding the gap between environmental intention and pro-environmental behavior towards the waste sorting and management policy of China. *International Journal of Environmental Research and Public Health, 18*(2). doi:10.3390/ijerph18020757

Ward, C., Holmes, G., & Stringer, L. (2018). Perceived barriers to and drivers of community participation in protected-area governance. *Conservation Biology, 32*(2), 437-446. doi:10.1111/cobi.13000

Wheeler, B. W., Lovell, R., Higgins, S. L., White, M. P., Alcock, I., Osborne, N. J., . . . Depledge, M. H. (2015). Beyond greenspace: An ecological study of population general health and indicators of natural environment type and quality. *International Journal of Health Geographics, 14*, 17. doi:10.1186/s12942-015-0009-5

White, R. G. (2020). Mental wellbeing in the Anthropocene: Socio-ecological approaches to capability enhancement. *Transcultural Psychiatry, 57*(1), 44-56. doi:10.1177/1363461518786559

White, R. L., Eberstein, K., & Scott, D. M. (2018). Birds in the playground: Evaluating the effectiveness of an urban environmental education project in enhancing school children's awareness, knowledge and attitudes towards local wildlife. *PLOS ONE, 13*(3), e0193993. doi:10.1371/journal.pone.0193993

Wickrama, K. A. S., & Wickrama, T. (2011). Perceived community participation in tsunami recovery efforts and the mental health of tsunami-affected mothers: Findings from a study in rural Sri Lanka. *International Journal of Social Psychiatry, 57*(5), 518-527. doi:10.1177/0020764010374426

Woodgate, R. L., & Skarlato, O. (2015). "It is about being outside": Canadian youth's perspectives of good health and the environment. *Health & Place, 31*, 100-110. doi:10.1016/j.healthplace.2014.11.008

Yessoufou, K., Sithole, M., & Elansary, H. O. (2020). Effects of urban green spaces on human perceived health improvements: Provision of green spaces is not enough but how people use them matters. *PLOS ONE, 15*(9). doi:10.1371/journal.pone.0239314

Young, N., Cooke, S. J., Hinch, S. G., DiGiovanni, C., Corriveau, M., Fortin, S., . . . Solås, A. M. (2020). "Consulted to death": Personal stress as a major barrier to environmental co-management. *Journal of Environmental Management, 254*, 109820. doi:10.1016/j.jenvman.2019.109820

Zhang, X., Zhang, Y., Zhai, J., Wu, Y., & Mao, A. (2021). Waterscapes for promoting mental health in the general population. *International Journal of Environmental Research and Public Health, 18*(22). doi:10.3390/ijerph182211792

Zhao, N., Li, B., Li, H., Li, G., Wu, R., Hong, Q., . . . Dong, R. (2021). The potential co-benefits for health, economy and climate by substituting raw coal with waste cooking oil as a winter heating fuel in rural households of northern China. *Environmental Research, 194*. doi:10.1016/j.envres.2020.110683

Zhao, Y., & Wise, N. (2019). Evaluating the intersection between "green events" and sense of community at Liverpool's Lark Lane Farmers Market. *Journal of Community Psychology, 47*(5), 1118-1130. doi:10.1002/jcop.22177

Appendix C: MMAT Criteria

Category of study designs	Methodological quality criteria
1. Qualitative	1.1. Is the qualitative approach appropriate to answer the research question?
	1.2. Are the qualitative data collection methods adequate to address the research question?
	1.3. Are the findings adequately derived from the data?
	1.4. Is the interpretation of results sufficiently substantiated by data?
	1.5. Is there coherence between qualitative data sources, collection, analysis and interpretation?
2. Quantitative randomized controlled trials	2.1. Is randomization appropriately performed?
	2.2. Are the groups comparable at baseline?
	2.3. Are there complete outcome data?
	2.4. Are outcome assessors blinded to the intervention provided?
	2.5 Did the participants adhere to the assigned intervention?
3. Quantitative non-randomized	3.1. Are the participants representative of the target population?
	3.2. Are measurements appropriate regarding both the outcome and intervention (or exposure)?
	3.3. Are there complete outcome data?
	3.4. Are the confounders accounted for in the design and analysis?
	3.5. During the study period, is the intervention administered (or exposure occurred) as intended?
4. Quantitative descriptive	4.1. Is the sampling strategy relevant to address the research question?
	4.2. Is the sample representative of the target population?
	4.3. Are the measurements appropriate?
	4.4. Is the risk of nonresponse bias low?
	4.5. Is the statistical analysis appropriate to answer the research question?
5. Mixed methods	5.1. Is there an adequate rationale for using a mixed methods design to address the research question?
	5.2. Are the different components of the study effectively integrated to answer the research question?
	5.3. Are the outputs of the integration of qualitative and quantitative components adequately interpreted?
	5.4. Are divergences and inconsistencies between quantitative and qualitative results adequately addressed?
	5.5. Do the different components of the study adhere to the quality criteria of each tradition of the methods involved?

Appendix D: Funding Sources for Included Studies

Article	Funding Details
Asah & Blahna (2012)	Funded by the U.S. Department of Agriculture Forest Service, Pacific Northwest Research Station.
Avon Wildlife Trust (2021)	National Lottery Community Fund; The Wildlife Trusts
Bellotti et al. (2011)	The VA Health Services Research and Development Postdoctoral fellowship program.
Birch (2005)	University of Brighton
Bond et al. (2019)	Not clear
Cardskadden & Lober (1998)	Duke University Nicholas School of Environment
Coventry et al. (2019)	University of York Research Priming Fund; UK Research and Innovation Closing the Gap Network.
Finnegan (2016)	Not clear
Fraser at al. (2009)	Institute of Museum and Library Services
Gagliardi et al. (2020)	Italian Ministry of Health; IRCCS INRCA
Gooch (2005)	University of Queensland & the Catchment Hydrology Cooperative Research Centre
Hoffman (2020)	Minnesota Agricultural Education Leadership Council
Hsiao et al. (2020)	No financial support received
Kogstad et al. (2014)	Not clear
Koss & Kingsley (2010)	Parks Victoria and Deakin University Research Partners Grant
Molsher & Townsend (2016)	State Natural Resource Management: Natural resources KI.
Moore et al. (2006)	Not clear
O'Brien et al. (2010)	Scottish Forestry Trust and Forestry Commission
O'Brien et al. (2011)	Part funded by the Scottish Forestry trust and the Forestry commission.
Pálsdóttir et al. (2014)	FORMAS
Pillemer et al. (2010)	Edward R. Roybal Center grant from the National Institute of Aging
Power & Smyth (2016)	Arts and Humanities Research Council
Puhakka et al. (2019)	TEKES
Reynolds (2000)	Not clear
Richardson et al. (2016)	Not clear
Richardson & McEwan (2018)	Not clear
Sobko et al. (2020)	General Research Grant
Takase et al. (2018)	Grants from JSPS KAKENHI
Tashiro (2022)	JSPS KAKENHI
Tharrey et al. (2020)	French National Research Agency; Agropolis Foundation; Olga Triballat Institute.
Townsend (2006; Study 1)	Parks Victoria, the People and Parks Foundation, Alcoa World Alumina Australia,
Townsend (2006; Study 2)	The Helen Macpherson Smith Trust, the Victorian Department of Sustainability and Environment,
Townsend (2006; Study 3)	The Trust for Nature, Barwon Health, Angair, Surf Coast Shire and the City of Hobsons Bay.
Weston et al. (2015)	World Vision
Wilkie & Michialino (2014)	Not clear
Wilson et al. (2009)	Forestry Commission Scotland; Glasgow Centre for Population Health; HSGGC; Glasgow Clyde Valley Green Network Partnership.
Yerrell (2008)	Oxford Brookes University
